# Improving Large‐Scale Population Estimates and Assessments of the Ecological Importance of Three Epifaunal Bivalve Species by Combining Distribution and Abundance Models

**DOI:** 10.1002/ece3.72586

**Published:** 2025-12-08

**Authors:** Youk Greeve, Molly C. Reamon, Per Bergström, Åsa Strand, Ane T. Laugen, Mats Lindegarth

**Affiliations:** ^1^ Department of Marine Sciences – Tjärnö University of Gothenburg Tjärnö Strömstad Sweden; ^2^ Department of Natural Sciences, Centre for Coastal Research (CCR) University of Adger Kristiansand Norway; ^3^ Department of Environmental Intelligence IVL Swedish Environmental Research Institute Fiskebäckskil Sweden

**Keywords:** mussels, oysters, SDM, Skagerrak, species abundance models, species distribution models, Sweden

## Abstract

Epifaunal bivalves include reef‐building organisms that provide several important ecological functions in coastal marine environments. Evaluating the distributional patterns and population sizes is key in assessing the total and relative contribution of species toward these functions and can aid in improving spatial planning and management. In this paper, the use of species distribution and abundance models as a means to improve population size estimates compared to more simplistic methods (i.e., extrapolation of mean densities) was investigated. Gradient boosting models were fitted to predict occurrences and densities of three, both native and invasive, ecologically important bivalve species (blue mussel, *Mytilus edulis* ; Pacific oyster, *Magallana gigas*; and European oysters, *Ostrea edulis* ) occurring on the Swedish west coast. Bootstrapping methods were used to estimate model performance and error margins around the final predictions of total population size. Additionally, the spatial predictions were used to extract information on the key habitat types of each species. Results from the distribution models show considerable overlap in the use of shallow habitat by the native blue mussels and the invasive Pacific oyster, while the European oyster resided mostly in deeper habitats. There were, however, differences in larger geographical distribution patterns among all species. While all distribution models performed adequately (AUC ≈ 0.75–0.9 and TSS ≈ 0.45–0.65), the largest difference in estimated population size was observed when involving abundance models, reducing them by ~70% and ~50% for blue mussels and Pacific oysters respectively, while the estimate for European oysters remained similar. Importantly, estimates of total abundance when translated to biomass estimates indicated that the invasive Pacific oyster likely contributes the most to ecosystem functions associated with epibenthic bivalves in this area (~50% of total estimated dry weight biomass).

## Graphical abstract

Epifaunal bivalves (e.g., mussels and oysters) include reef building organisms that provide several important ecological functions in coastal marine environments. Using Gradient Boosting Machine models, the distribution and abundance patterns of three important species were modeled on the Skagerrak coast of western Sweden. The combined output of these models allowed evaluation of the habitat use overlap and differences, as well as improving estimates of total population size and relative contribution to ecosystem functions among species.

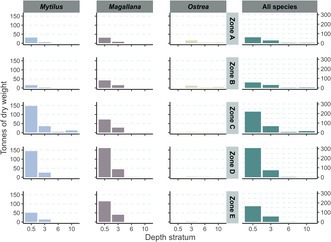

## 1 Introduction

Epifaunal bivalves (e.g., mussels and oysters) are key components of many coastal ecosystems. They are often very abundant and have ecosystem‐engineering properties that play critical roles in the functioning of ecosystems and support several ecosystem services (zu Ermgassen et al. 2020). Firstly, they often congregate into dense assemblages, so‐called biogenic reefs, that provide structural habitat for other species and promote biodiversity (Kristensen et al. 2015; Norling et al. 2015). Secondly, as suspension feeders, they capture phytoplankton and other particles, thereby regulating plankton communities (Grabowski et al. 2012) and increasing water clarity (Peterson and Heck 2001; Newell 2004). At the same time, they transport excess nutrients to the seabed through their feces where they can be further decomposed by micro‐organisms and sequestered (Newell et al. 2002; Caffrey et al. 2016). Bivalve reefs have been dramatically reduced on a global scale in recent history because of over‐exploitation and habitat destruction, among other causes (Beck et al. 2011). The growing concern for the health of marine systems in turn has sparked many conservation efforts to restore natural bivalve populations in areas where reefs were once a defining feature (Smith and Pruett 2024).

On the west coast of Sweden, there are three species of epifaunal bivalves dominating the community structure in shallow coastal areas (Greeve et al. 2023); the blue mussel ( *Mytilus edulis* , from here on: *Mytilus*), the invasive Pacific oyster (*Magallana gigas*, previously *Crassostrea gigas* , from here on: *Magallana*), and the European flat oyster ( *Ostrea edulis* , from here on: *Ostrea*). All three of these species perform similar functions within the coastal ecosystems but also differ in their ecology. Like in other regions in the northeast Atlantic (Sorte et al. 2017), the extent of *Mytilus* reefs on the Swedish west coast is perceived to have declined dramatically in recent decades (trends and potential causes reviewed in Baden et al. 2021; Laugen et al. 2023). During the same period, *Magallana* has recently (2006) successfully invaded and expanded its distribution throughout the west coast region (Wrange et al. 2010; Laugen et al. 2015). *Ostrea* is functionally extinct throughout most of its original distribution (Helmer et al. 2019; Thurstan et al. 2024), but recent monitoring has demonstrated that a substantial population has persisted on the Swedish west coast (Thorngren et al. 2019). Although the functional roles in ecosystems of these species have been widely studied mainly on individuals, there is limited information on the extent to which they utilize different habitats and overall population sizes due to a lack of monitoring and mapping. Characterizing the population structure of each of these species can resolve how they compare in terms of ecological significance, how they are likely to interact with each other, as well as monitor changes in the future.

Species distribution models (SDMs) are numerical tools used to predict the distribution of species (Guisan and Zimmermann 2000). Observations of species (e.g., presence or absence) are combined into maps of relevant explanatory variables to produce models, which are then used to project the expected occupied range across a certain area. Examples of common applications of SDMs include predicting changes in a species' range under a changing climate (Brun et al. 2020; Santini et al. 2021) or providing a basis for assigning critical areas for conservation efforts (Guisan et al. 2013). For many ecological questions, modeling the abundance of species in expected occupied habitats is more appropriate since many ecological processes are related to a species' density. Species abundance models are, however, far less frequently used by scientists in marine settings compared to SDMs (Melo‐Merino et al. 2020), despite their potential to enhance the value of the projection for many applications. This can, to some degree, be explained by the often sub‐optimal performance of such abundance models, especially when predicting in unsampled areas or under new climate conditions (Waldock et al. 2022).

Thus, the overall aim of this study was to assess the occurrence, abundance, and ecological importance of *Mytilus*, *Magallana*, and *Ostrea* in Swedish coastal waters using SDM and abundance modeling. An extensive and recently collected data set consisting of a total of 796 sampled sites was used to develop models to predict the spatial distribution and local abundances of the three target species at a high resolution. The models were used to unravel correlative patterns for each species and various environmental factors, to predict and estimate local and total population sizes, as well as evaluate habitat preferences for each species. Ultimately, predicted abundances and additional data on biomass were used to assess the likely relative contribution of each species to overall ecosystem functions.

## 2 Methods

### 2.1 Study Area and Sampling

**FIGURE 1:**
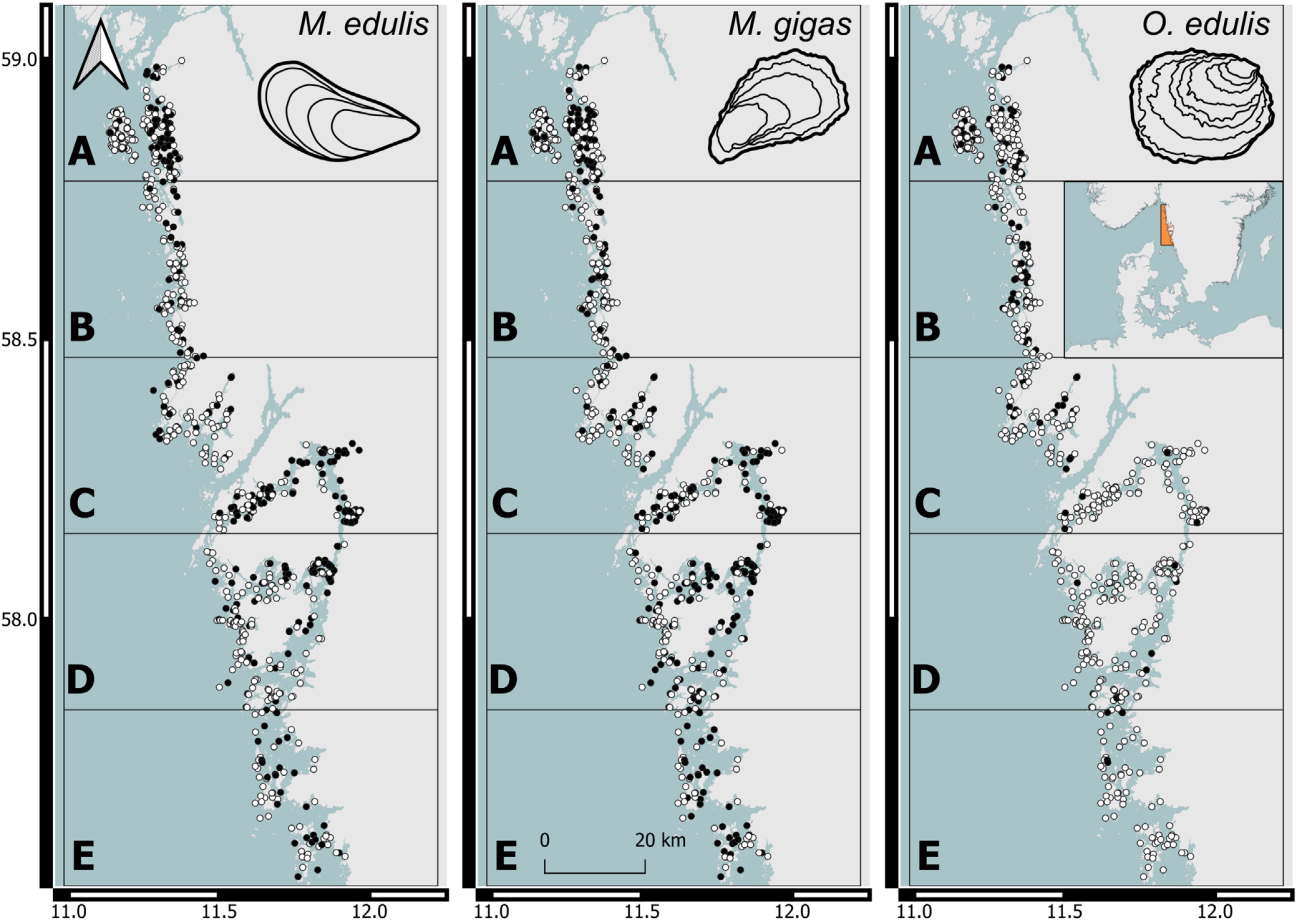
The study area and sample locations for each species (*Mytilus* (*edulis*): Blue mussel, *Magallana* (*gigas*): Pacific oyster, *Ostrea* (*edulis*): European flat oyster) as dots with presences in black and absences in white. The zones used for estimating population sizes in Method 1 and 2, and for visualization of results are labeled on the left (A–E).

This study was based on data collected on the Swedish part of the Greater North Sea coast between the Norwegian border in the north and Gothenburg in the south (Figure 1). This area is geographically diverse, with several archipelagic regions (characterized by numerous islands of different sizes) and several fjords (large and narrow inlets). These characteristics imply substantial variability in oceanographic conditions on small spatial scales. Consequently, very different environments ranging from wave‐battered rocks to silty bays occur in close proximity. The tides are marginal, with a tidal range of only about 30 cm, though high and low water of > 1 m occur occasionally (Johannesson 1989).

Data on bivalve occurrence and abundance were compiled from a number of surveys conducted between 2018 and 2023, with varying spatial cover and methods (Table S1). All studies targeted either one or all of *Mytilus*, *Magallana*, and *Ostrea*, and locations were either completely randomized or randomized within strata of depth and wave exposure. Parts of the data were collected using camera tows, where a GoPro camera was attached to a sled and dragged along the isobath behind a small boat in two transects (each 20 m × 0.8 m = 16 m^2^, see Thorngren et al. 2019 for sampling method details). The footage was analyzed, and the number of individuals of each species counted. Other parts of the data were collected using 5 or 10 replicate quadrates (usually 1 m^2^, but occasionally 0.25 m^2^ when high densities were so high that subsampling was required) along a 40–100 m transect in the intertidal and shallow subtidal zone, collecting and counting all individuals by hand. Both methods were compared within the same location, validating the accuracy and similarity of results for all three species (Reamon et al. 2024), thus biases introduced by variable counting methods in different depth strata were unlikely. In most instances, the percentage cover of various substrate classifications (mud, sand, gravel, rock, etc.) was estimated visually. In total, 796 surveyed sites were included in the study, of which 555 sites were surveyed using camera tows (at depths of 0.5–10 m) and 241 sites were surveyed using quadrats (at depths of 0–2 m, Figure 1).

The 796 sites were well distributed along the coastline, although the Gullmar fjord was never incorporated in any of the surveys. There was also a higher concentration of sites in the northern area (Figure 1). *Mytilus*, *Magallana*, and *Ostrea* were recorded at 280 (35%), 314 (39%), and 130 (16%) sites, respectively. At sites where the species were present, the mean densities (individuals m^−2^) were 22.9, 4.8, and 0.7, respectively (Table S2).

### 2.2 Empirical Modeling

All data handling, modeling, and visualizations were done using purpose‐built scripts in R v. 4.2.2 (R Core Team 2022) and libraries specific to individual tasks (see below).

#### 2.2.1 Predictor Variables

To develop predictive models of the occurrence and abundance of bivalves, physical and chemical predictor variables reflecting the bathymetry and conditions in the benthic and pelagic environments were used. GIS layers of predictive variables were extracted from various sources and based on either mapped bathymetry, oceanographic models, or satellite imagery (Table S3). Variables were selected based on their availability, resolution, cover of the study area, and ecological relevance for the modeled species, based on personal experience or scientific literature (e.g., Snickars et al. 2014). Because depth was expected to be one of the most important variables (Bergström et al. 2021), it was kept at the highest resolution possible (10 × 10 m). Variables with coarser resolutions (ranging from 25 × 25 m to 2 × 2 km, see Table S3) were resampled to this resolution using nearest‐neighbor interpolation. Since the high‐resolution satellite‐derived depth data had incomplete coverage (Huber et al. 2022), it was partially filled in using resampled lower‐resolution bathymetry‐based depth data (Albertsson et al. 2006). The variables coastal position (*Y*) and in‐offshore position (*X*) were created by rotating the coordinates (reference system: SWEREF99, units in meters) by 14° to compensate for the inclination of the shoreline and rescaling them using min‐max normalization. This resulted in an *XY*‐coordinate system that retained all information of distances but better represented the along shore (south to north) and in‐ offshore (east to west) gradients across the entire study area, which were of more ecological interest. Several alternative aggregations (mean, median, maximum, and minimum) of the monthly interpolated means of satellite‐derived layers (i.e., turbulence, suspended particulate material, and chlorophyll; CMEMS 2024a) and mean daily surface values derived from oceanographic models (i.e., salinity and temperature; CMEMS 2024b) were made from the years 2021 to 2023.

Multicollinearity between variables can introduce inaccuracies in SDMs (De Marco Jr and Nóbrega 2018), and thus all variables were screened and removed stepwise (highest removed) based on their VIF (variance inflation factor) until the commonly used conservative minimum of 5 was achieved (Guisan and Zimmermann 2000). Next, variables with the highest VIF score that were already represented in the variable list by a related measure (minimum, maximum, median, or mean) were removed. This resulted in 13 variables left after the screening process: depth, slope, exposure, distance, Y, X, satellite‐derived submerged aquatic vegetation (SAV), proportion hard substrate (*P*_HARD_, see section 2.2.3), and proportion soft sediment substrate (*P*_SOFT_, see Section 2.2.3.), maximum chlorophyll (Max.Chl), minimum salinity (Min.Sal), maximum suspended particulate material (Max.SPM), and median temperature (Med.Temp), which were used in the models (Table S3).

#### 2.2.2 Model Training and Uncertainty

Randomly splitting the data once into training‐ and test‐data (e.g., 80% for training and 20% for evaluation) is common practice in model training (Austin 2007). However, model performance can vary substantially based on the random composition of these data sets, potentially leading to false confidence in the models' predictions. Instead, all model parameters and performance metrics in this study were estimated using the bootstrap 0.632+ method (Efron and Tibshirani 1997; Potts and Elith 2006). This method can be used to produce near‐unbiased estimates of any performance metric by fitting models on many repeated bootstrap samples of the data and measuring performance on the omitted data. Thus, 100 bootstrap samples were taken from the whole dataset, used to fit models, estimate model parameters (e.g., constants, variable importance, and cut‐off thresholds), and various performance metrics (e.g., AUC, *r* ^2^). The results from the 100 bootstrap iterations were then combined to estimate the performance of a model that included all available data, thus making maximum use of data while at the same time minimizing random variation in the model performance and accounting for overfitting (Efron and Tibshirani 1997). For a simplified guide to the bootstrap 0.632+ approach, see Potts and Elith (2006).

#### 2.2.3 Substrate Models

Since no comprehensive and accurate high‐resolution maps on substrate in shallow Swedish coastal waters exist, observations from the field data were used to model and predict the cover of various substrate types. From the field studies, 749 sites (out of 796) in the data set had visually estimated percentages of local substrate conditions. These models were used to predict the cover of hard substrate (sum of non‐mobile rock and stones, from here on: “*P*_HARD_”) and the cover of soft sediment (proportion of muddy sediment: “*P*_SOFT_”). The latter was calculated as the percentage of muddy sediment only considering mobile fractions (i.e., after excluding “*P*_HARD_”). This was done to reflect the fact that historical geological processes may have determined the cover of rocky substrate while ongoing wave action and currents are continuously modifying the cover of mobile fine or coarse sediments. This means that extremely erosive and mobile sediments can occur side by side with stable rocky substrate in the same site, and both are a result of very different processes. The coarse substrate (e.g., gravel and shell hash) was not modeled since it was already implied by being the remaining fraction beside the combination of “*P*_HARD_” and “*P*_SOFT_”.

Boosted generalized additive models (BGAM) were fitted to predict the cover of *P*_HARD_ and *P*_SOFT_ using the “gamboostLSS” package (Hofner et al. 2014, v.2.0.6). The variables used in the substrate models were limited to those of a more physical nature (depth, slope, exposure, distance, *Y*, *X*, and SAV). As the proportions of substrate had a distribution between 0 and 1, with over‐inflation of both 0 and 1, a beta inflation distribution was chosen to fit the models. The learning rate was set to 0.05, and a noncyclical tuning method was used to optimize the boosting process (Thomas et al. 2018). The models were then projected over the study area using the raster layers of the used variables at a 10 × 10 m resolution.

#### 2.2.4 Presence–Absence Models

SDMs of presence–absence of all three species were fitted using gradient‐boosted tree models (GBM) using the “caret” package (Kuhn et al. 2011, v6.0.93). Machine learning algorithms, such as GBM, often outperform statistical models when applied as SDMs. They provide several advantages when working with large datasets including many variables, such as the ability to fit complex relationships with explanatory variables and the interactions between them (Elith et al. 2006). The Receiver Operating Characteristic Area Under the Curve (from here on: AUC) score, which is the most used metric for evaluating classification model performance (Jiménez‐Valverde 2012), was chosen as the metric to optimize in the boosting process. To interpret the performance, however, we used a wider selection of metrics: specificity, sensitivity, accuracy, and true skill statistic (TSS). The models were tuned for shrinkage, number of trees, and the minimum observations per node, after which the model was fitted for each bootstrap sample of the data using repeated cross‐validation (*k* = 10, *n* = 10).

Several approaches exist to translate predicted probabilities of occurrence into presences and absences (Nenzén and Araújo 2011; França and Cabral 2019). One commonly used approach is to optimize the discriminatory power of the model by maximizing the true positives and the true negatives by setting a cut‐off threshold between presences and absences (i.e., using the Youden index). To avoid issues with overestimation of a species distribution, which is common when using the Youden index for species with low prevalence (Smits 2010), a cut‐off threshold was set to adjust modeled prevalence to match that of the observed data. This was deemed appropriate as stratified random sampling campaigns were used in this study, which means that the observed prevalence can be expected to estimate the true prevalence (compared to a more subjective selection of sampling sites). This process was replicated for each bootstrap iteration and the resulting cut‐off threshold values were averaged and used for the final predictions.

#### 2.2.5 Abundance Models

The modeling to predict abundances of the three species was done in a similar way to that of the presence–absence models. The same predictor variables were used, but the average of the out‐of‐bag presence–absence model predictions in each bootstrap iteration (non‐binary probability of occurrence) of each species was also included as predictor variables for each species respectively. Prior to model fitting, sites where the modeled species were not recorded were removed from the data set and the abundances were converted to density (ind. m^−2^) by dividing the counted individuals at each sample site by the sampled area. GBMs were again used to fit regression models with the same tuning protocol as the presence–absence models (see Section 2.2.4), this time using root mean square error (RMSE) as the optimisation metric. The densities were log‐transformed prior to model fitting, as initial trials suggested this improved model performance. The model predictions were back‐transformed to regular densities for estimating the error metrics.

Inconsistent bias resulting in over‐prediction at low values and under‐prediction at high values is a common occurrence in species abundance (Potts and Elith 2006) and machine‐learning regression models (Belitz and Stackelberg 2021). A post hoc adjustment can be applied to model predictions to improve the fit between predicted and observed values. Therefore, a regression of observed on estimated values (ROE) correction was applied to improve the model fit, followed by Duan's smearing estimate (Duan 1983) to account for the back‐transformation of the log‐transformed values (Belitz and Stackelberg 2021). The corrections for the final model were done by averaging the correction parameters of the bootstrap iterations. The predicted densities on the entire study area were adjusted and back‐transformed and were multiplied by the areal extent (10 × 10 m = 100 m^2^) of each raster cell to calculate the predicted number of individuals per cell.

### 2.3 Comparing Niches and Relative Importance of Species

#### 2.3.1 Importance of Predictor Variables for Individual Species

Differences in habitat preferences and the niche of *Mytilus*, *Magallana*, and *Ostrea* were inferred from inspection of the scaled (0–100) variable importance scores and the partial response curves extracted from the models using all available data. The produced presence–absence and abundance maps were subsequently used to assess the quantitative contributions of individual species in terms of occurrence and abundances in different parts of the study area and in particular types of habitats. To keep computation times reasonable, a large subsample (*n* = 10,000 cells) of the maps (a total *N* ≈ 7 million cells) was taken together with the corresponding variables and used to explore general patterns. As depth was expected to influence the distribution and potentially abundance, the subsamples were grouped into depth strata using intervals of 0.5 m. Similarly, a division was made between “sheltered” (exposure < 10^4^) and “exposed” (exposure > 10^4^) areas, as this division has previously been used and found to result in meaningful differentiation of habitat types in terms of substrate and corresponding differences in epifaunal bivalve community structure (Greeve et al. 2023).

#### 2.3.2 Population Size and Ecological Relative Importance of Species

Estimates of the total population (i.e., all individuals residing within the study area) sizes were calculated for each species to explore potential differences in relative contribution to ecosystem functions and services between species. This was done using three alternative methods with increasing complexity.

Method 1 was a simplistic model based on multiplying mean observed densities (using both presences and absences) over the total area of several depth strata (0–0.5, 0.5–3, 3–6, and 6–10 m) within 5 zones (Figure 1A–E). Segregating the extrapolation into several zones improves the total estimate because the distribution and average density vary from north to south. More segmentation increases accuracy if there are sufficient samples remaining within each zone. To balance this trade‐off, the coastline was divided into 5 equally sized zones. This method was spatially implicit and, in essence, replicates the methods (including the same depth strata and the use of zones) used for the population calculation for *Ostrea* previously performed on the Swedish west coast (Thorngren et al. 2019), in turn based on methods described by Cochran (1977), but within a larger area and with the inclusion of the 0–0.5 m stratum in this study.

Method 2 utilized information from the presence–absence models by extrapolating the mean observed densities (when present) multiplied by the area that was predicted to be occupied (for each generated cut‐off threshold value) in each depth strata and zone. The added uncertainty introduced by the variation in cut‐off thresholds was incorporated through propagation of error. Finally, Method 3 combined the results of the presence–absence and abundance models. The raster layer from the abundance model predictions was delimited by the cut‐off threshold from each bootstrap iteration and the total sum of individuals was calculated. Next, the average of these sums was divided by the average ratio between the sum of predicted and observed (omitted data) individuals of each abundance model bootstrap iteration. This was done to represent the uncertainty in the abundance models performance in estimating a population size, as well as correcting for consistent biases in doing so. Finally, because ecological functions, such as production and filtration, are linked to biomass rather than abundance (Riisgård 2001), the total population size was multiplied by a species‐specific average dry weight (DW) and live wet weight (WW) biomass to transform aggregated abundances into generalized estimates of total biomass (DW and WW measurements taken from Greeve et al. 2023). See the supplement for details on all calculations.

## 3 Results

### 3.1 Substrate Model Performance

**FIGURE 2:**
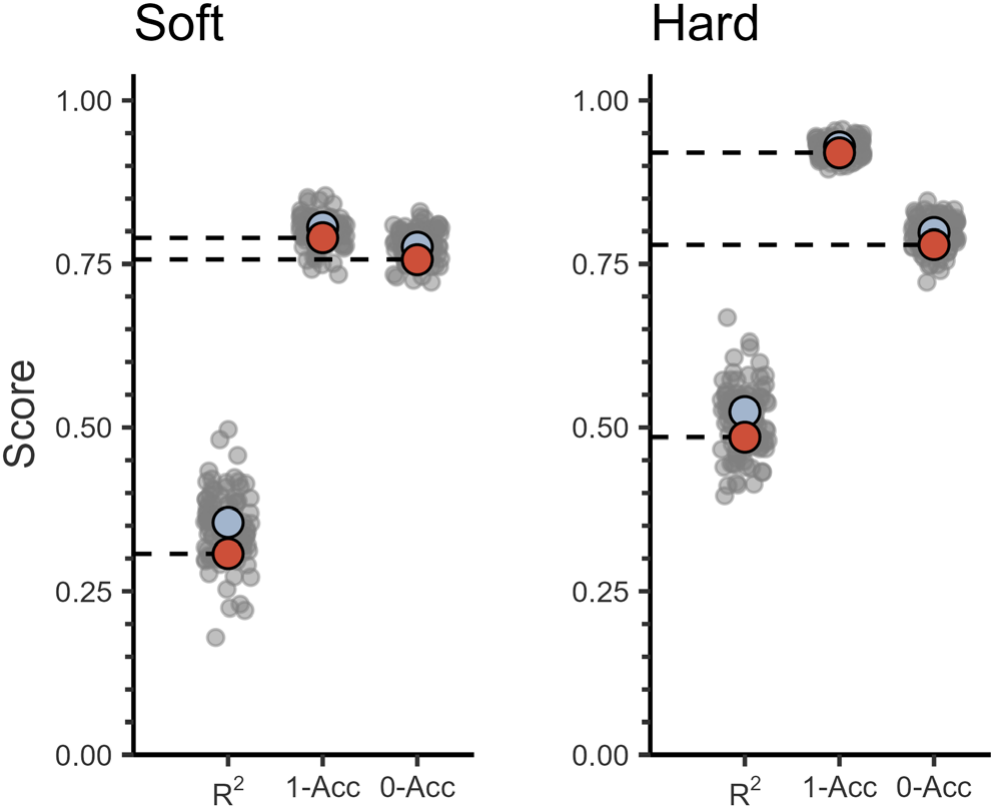
Model performance scores of soft and hard substrate cover models (boosted generalized additive models, BGAMs). Performance metrics are displayed on the x‐axis. 1/0‐Acc scores are the accuracy of predicting either 100% or 0% cover of each substrate type. Apparent performance represents the fit of all available data to its own training data (blue dots). Bootstrap performance represents the fit of each individual bootstrap iteration (gray dots). Best estimate (red dots) is the adjusted performance calculated from the bootstrap 0.632+ method.

Boosted generalized additive models (BGAM) were used to predict the percentage of hard and soft substrates, *P*_HARD_ and *P*_SOFT_. The optimal number of boosting iterations that minimized the risk of overfitting was 866 for *P*_SOFT_ and 1513 for *P*_HARD_. The estimated explanatory power of the models for *P*_SOFT_ was *R* ^2^ = 0.31, and the accuracy for predicting no cover (i.e., *P*_SOFT_ = 0%) was 0.76, and for full cover (i.e., *P*_SOFT_ = 100%) was 0.79 (Figure 2). The model of *P*_HARD_ performed better with an estimated *R* ^2^ = 0.49 and an accuracy for predicting no cover of 0.78 and for full cover of 0.92. The bootstrapping showed that estimates of accuracy were generally more precise than estimates of predictive power. Overall, there was a small difference between the average performance of the fitted models (“apparent”) compared to that of the average of external validations (“best estimate”). Thus, the models were deemed informative enough to use for the presence–absence and abundance models.

### 3.2 Presence–Absence Model Performance

**FIGURE 3:**
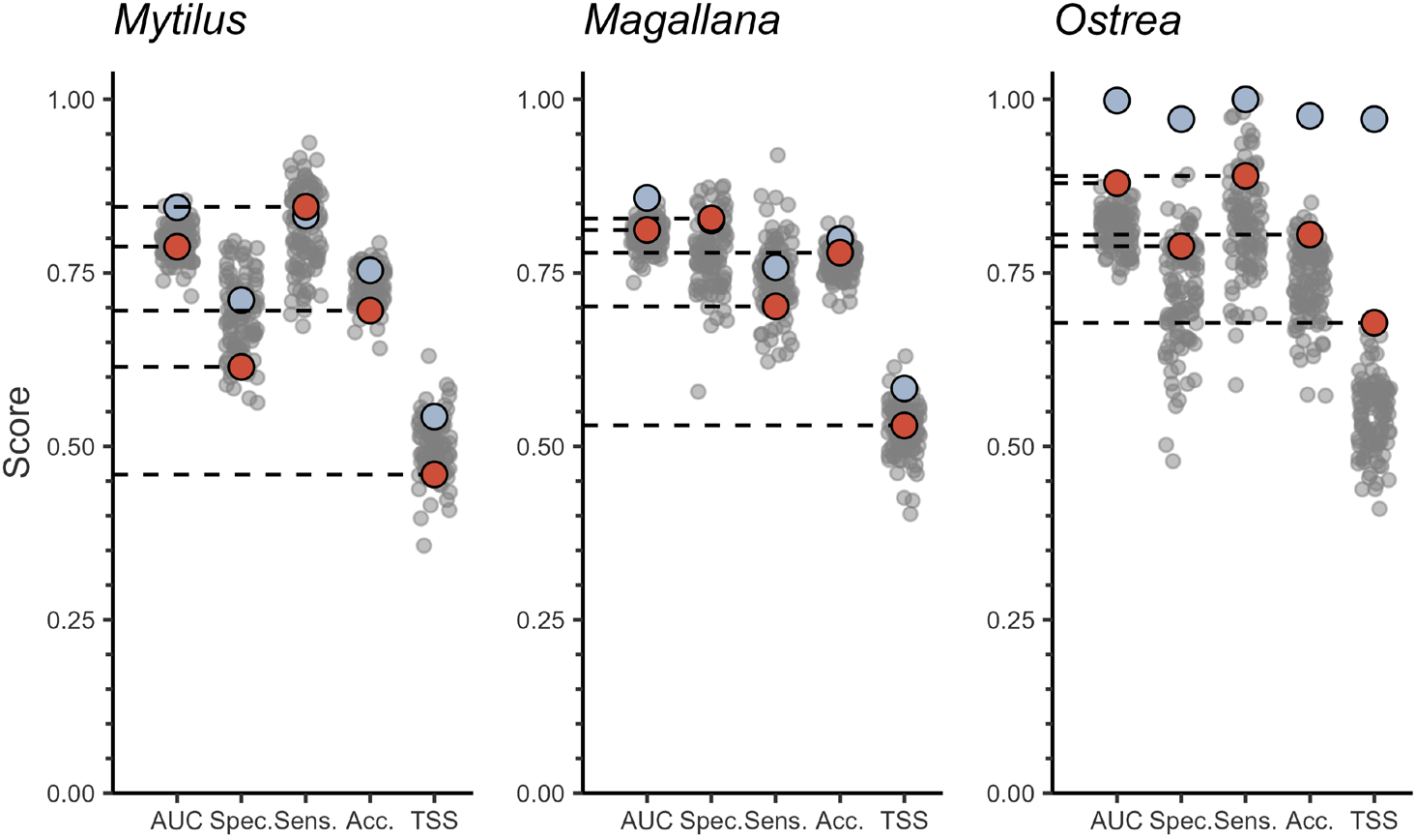
Model performance scores of presence–absence models (gradient‐boosted tree models, GBMs) for each species (*Mytilus* (*edulis*): Blue mussel, *Magallana* (*gigas*): Pacific oyster, *Ostrea* (*edulis*): European flat oyster). Performance metrics are given on the x‐axis (AUC = area under the curve, Spec. = specificity, Sens. = sensitivity, Acc. = accuracy, TSS = true skill statistic). Apparent performance represents the fit of all available data to its own training data (blue dots). Bootstrap performance represents the fit of each individual bootstrap iteration (gray dots). Best estimate (red dots) is the adjusted performance calculated from the bootstrap 0.632+ method.

**FIGURE 4:**
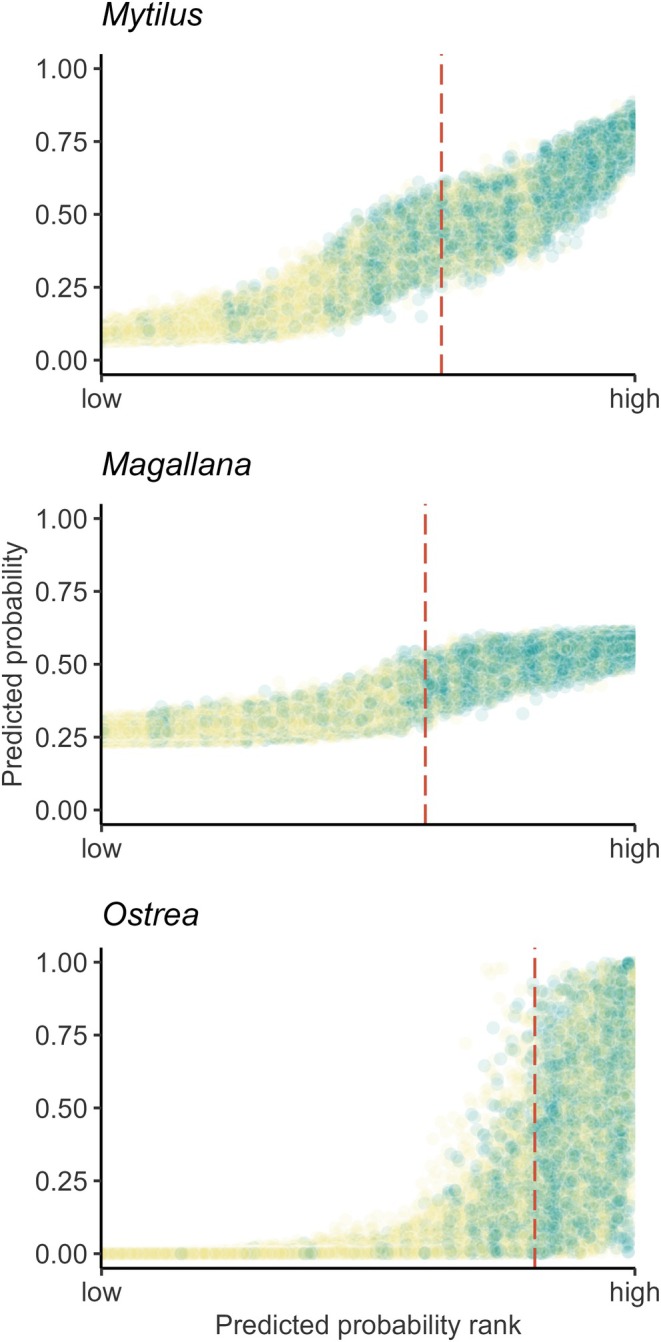
Visual inspection of the fit of the presence–absence models (gradient‐boosted tree models, GBMs) against observations for each species (*Mytilus* (*edulis*): Blue mussel, *Magallana* (*gigas*): Pacific oyster, *Ostrea* (*edulis*): European flat oyster). Data points are ranked on their average predicted probability from lowest to highest. The true observed presence (green dots) or absence (yellow dots) is indicated by color. The dashed red line indicates the cut‐off threshold calculated as the average of cut‐off threshold values produced in all bootstrap iterations.

The “best estimates” for AUC scores were 0.79, 0.81, and 0.88 for *Mytilus*, *Magallana*, and *Ostrea*, respectively (Figure 3). This indicates that the models of occurrence of all species were informative (Araújo et al. 2005). The relative performance in terms of sensitivity versus specificity differed among the species. Models of *Mytilus* and *Ostrea* were generally more sensitive (i.e., better at finding true positives), while models of *Magallana* were more specific (i.e., better at finding true negatives). The apparent scores for the *Ostrea* model were notably higher than the out‐of‐bag scores, indicating this model had more problems with overfitting, though the corrected best estimates still indicated meaningful performance (Araújo et al. 2005). The bootstrap distributions also show that the estimates of performance for the *Ostrea* model had substantially larger errors than for the other species. Thus, it was evident that models for all three species were generally capable of identifying species presences and absences correctly, though there was more disagreement between bootstrap iterations for *Ostrea*, particularly in sites with higher probability of presences (Figure 4).

### 3.3 Abundance Model Performance

**FIGURE 5:**
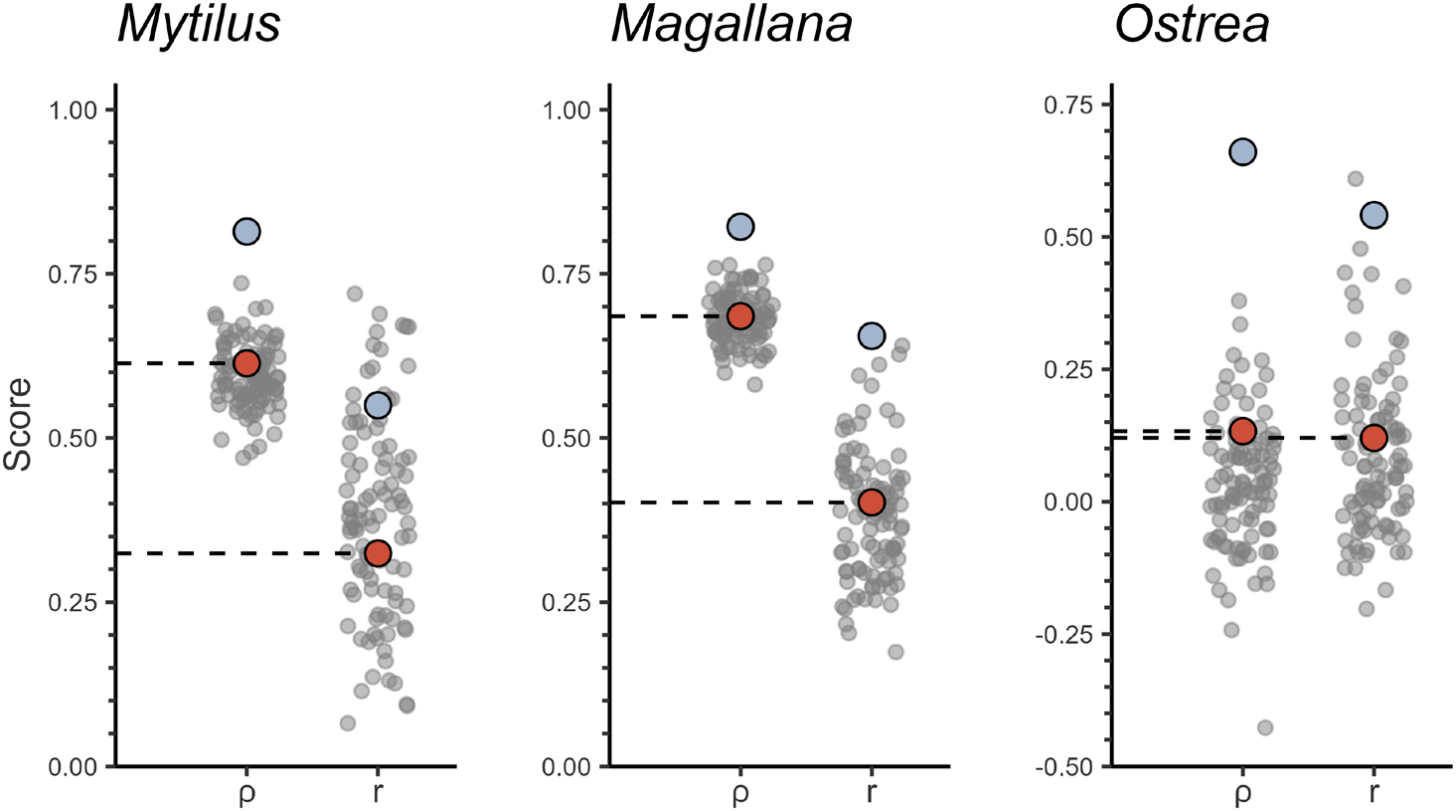
Model performance of the abundance models (gradient‐boosted tree models, GBMs) for each species (*Mytilus* (*edulis*): Blue mussel, *Magallana* (*gigas*): Pacific oyster, *Ostrea* (*edulis*): European flat oyster) calculated at an untransformed scale. Performance metrics are given on the *x*‐axis (*ρ* = Spearman's rank correlation coefficient, *r* = Pearson correlation coefficient). Apparent performance represents the fit of all available data to its own training data (blue dots). Bootstrap performance represents the fit of each individual bootstrap iteration (gray dots). Best estimate (red dots) is the adjusted performance calculated from the bootstrap 0.632+ method. Note the *y*‐axis difference for Ostrea.

**FIGURE 6:**
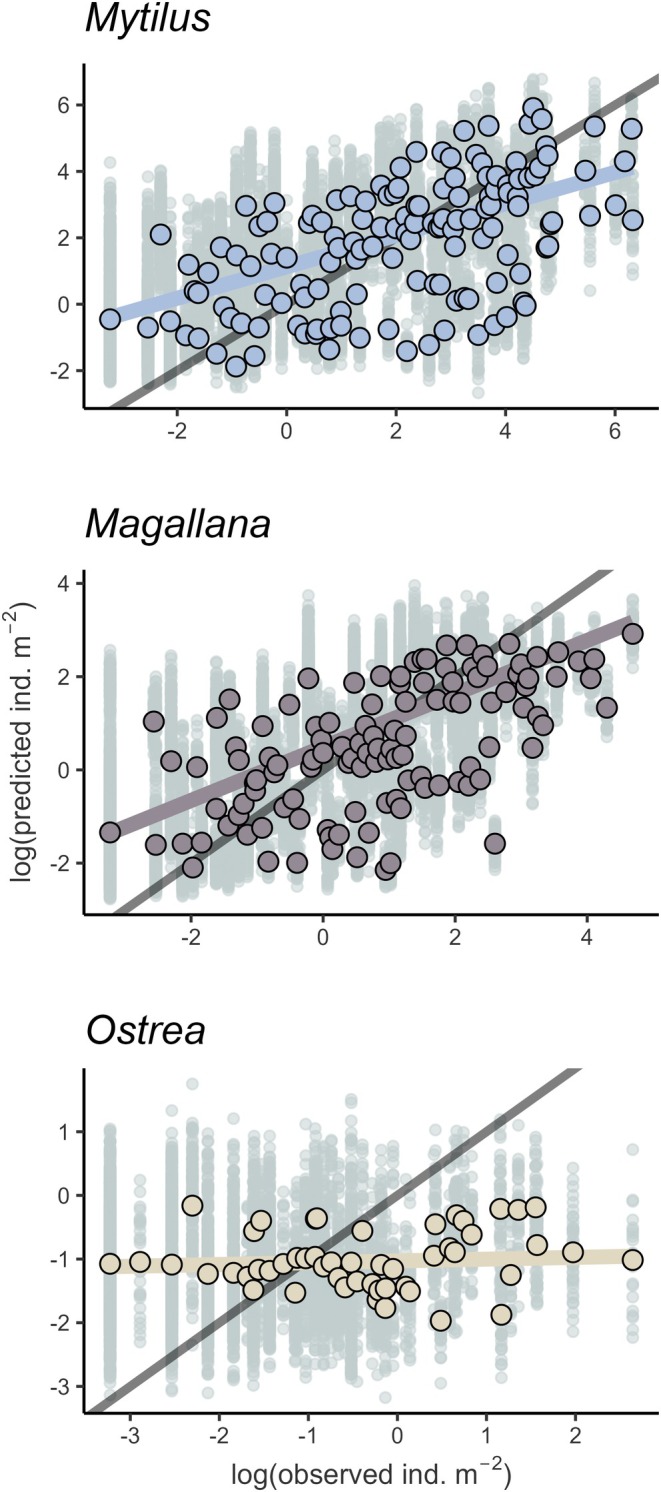
Average biased corrected observed versus predicted abundances (individuals m‐2) of each species (*Mytilus* (*edulis*): Blue mussel, *Magallana* (*gigas*): Pacific oyster, *Ostrea* (*edulis*): European flat oyster) from the abundance models (gradient‐boosted tree models, GBMs) on a logarithmic scale. Bootstrap prediction for each data point is given in gray. Dark gray line indicates the perfect (1 to 1) fit. Colored lines are the linear relations between the observed and the (average) predicted abundance.

The performance of the abundance models was also variable between species. The estimate of the Spearman rank coefficient (*ρ*) of the *Mytilus* and *Magallana* (0.61 and 0.69, respectively) indicated that the models were able to distinguish between low‐ and high‐abundance sites, though this did not seem to be the case for *Ostrea*, which had a lower ρ (0.13) with a high degree of variability between bootstrap iterations (Figure 5). The strength of quantitative relationships between predicted and observed abundances expressed as the Pearson's correlation coefficient (*r*) and the corresponding *R* ^2^ score were “moderate” (Schober and Schwarte 2018) for *Mytilus* and *Magallana* (*r* = 0.32 and 0.4, respectively). Again for *Ostrea*, the correlation was much lower (*r* = 0.12). Significant correlations between both untransformed and log‐transformed average predictions and observed densities for *Mytilus* and *Magallana* (untransformed *r* = 0.38) indicated that these models were, on average, informative (Figure 6). Despite the applied bias corrections, the median predictions for *Mytilus* and *Magallana* still consistently over‐predicted low and under‐predicted high abundances.

### 3.4 Variable Importance and Partial Dependence

Across all models, depth was the first or second ranked variable in variable importance, highlighting its effect on both distribution and abundance patterns (Table 1). The only exception was for the presence–absence of *Ostrea*, where *Y* (coastal position) was the most important variable, indicating that the distribution along the coast was particularly pronounced. *X* (in–offshore position) was ranked second for both model types of *Mytilus* and the presence–absence of *Magallana*, indicating there was a strong disparity for the distribution of these species and the abundance of *Mytilus* across this gradient. The modeled substrates (*P*_Hard_ and *P*_Soft_) were not very important for any of the models except for the abundance of *Magallana* (*P*_Hard_, ranked second). *P*_Presence_ (modeled probability of presences) was not overly important for any of the abundance models, suggesting that the mechanisms that determine distribution and abundance patterns were inherently different.

**TABLE 1:** Scores of variable importance for the variables used in presence–absence and abundance models for each bivalve species (*Mytilus* (*edulis*): Blue mussel, *Magallana* (*gigas*): Pacific oyster, and *Ostrea* (*edulis*): European flat oyster).

Type	Variable	*Mytilus*		Magallana		Ostrea	
		Pres/Abs	Abund.	Pres/Abs	Abund.	Pres/Abs	Abund.
GIS‐data	Depth	**100**	**100**	**100**	**100**	**95.5**	**100**
	Distance (from shore)	4.9	9.9	2.9	7.0	50.7	**42.5**
	Exposure	8.5	9.1	3.5	2.5	50.9	11.4
	*Y*	3.2	9.3	9.6	8.5	**100**	32.8
	*X*	**61.3**	**25.8**	**24.7**	4.0	35.9	20.8
	Slope	7.9	13.8	3.2	8.4	38.2	22.8
Satellite data	Max.Chl	4.2	11.4	**18.8**	5.0	**64.8**	19.5
	Min.Sal	1.4	7.9	2.9	3.7	**60.3**	13.5
	SAV	1.6	0.6	0.3	0.2	3.4	2.7
	Max.SPM	5.1	7.2	2.4	6.8	47.7	**57.5**
	Med.Temp	4.8	8.3	2.1	5.2	30.7	6.6
Modeled data	*P*_HARD_	0.2	4.8	3.7	**13.0**	15.9	1.4
	*P*_SOFT_	3.4	7.7	2.5	5.4	27.3	20.4
	*P*_presence_		**18.1**		6.5		13.7

*Note:* The scores are scaled so the most important variable always has the maximum value of 100. Bold and shaded values indicate variables of notable importance for their respective models.

Abbreviation: SAV, submerged aquatic vegetation.

Partial response curves showed some contrasting patterns between species (Figures S1 and S2), though the relevance of these also depended on the variable importance. Most notably, for “depth” a strong positive response for presences was observed in the shallowest depths for *Mytilus* and *Magallana*, while there was an opposite trend for *Ostrea* (Figure S1). A similar contrast was visible for both *X* and exposure, suggesting that exposed habitat favored *Ostrea* more than the other species. The partial response curves for the abundance models showed a peak for *Ostrea* around 2 m depth, while for *Mytilus* and *Magallana* it increased more toward 0 m (Figure S2). Moreover, as indicated by the response curves for X, all species decreased in abundance toward more inshore habitats, though *Mytilus* was the only one to increase in abundance the further away it was from any landmass. There was a strong response from all species to *P*_Hard_ and *P*_Soft_, indicating that medium to high cover of rock and medium to low cover of soft (i.e., not gravel or shell hash) substrate favored higher abundances. Max.SPM (maximum suspended particulate matter), the second most important variable for the *Ostrea* abundance model, showed that a low suspended particulate matter content of the water had a positive effect on the abundance of this species.

### 3.5 Predicted Spatial Distributions and Population Sizes

#### 3.5.1 Patterns of Occupancy

**FIGURE 7:**
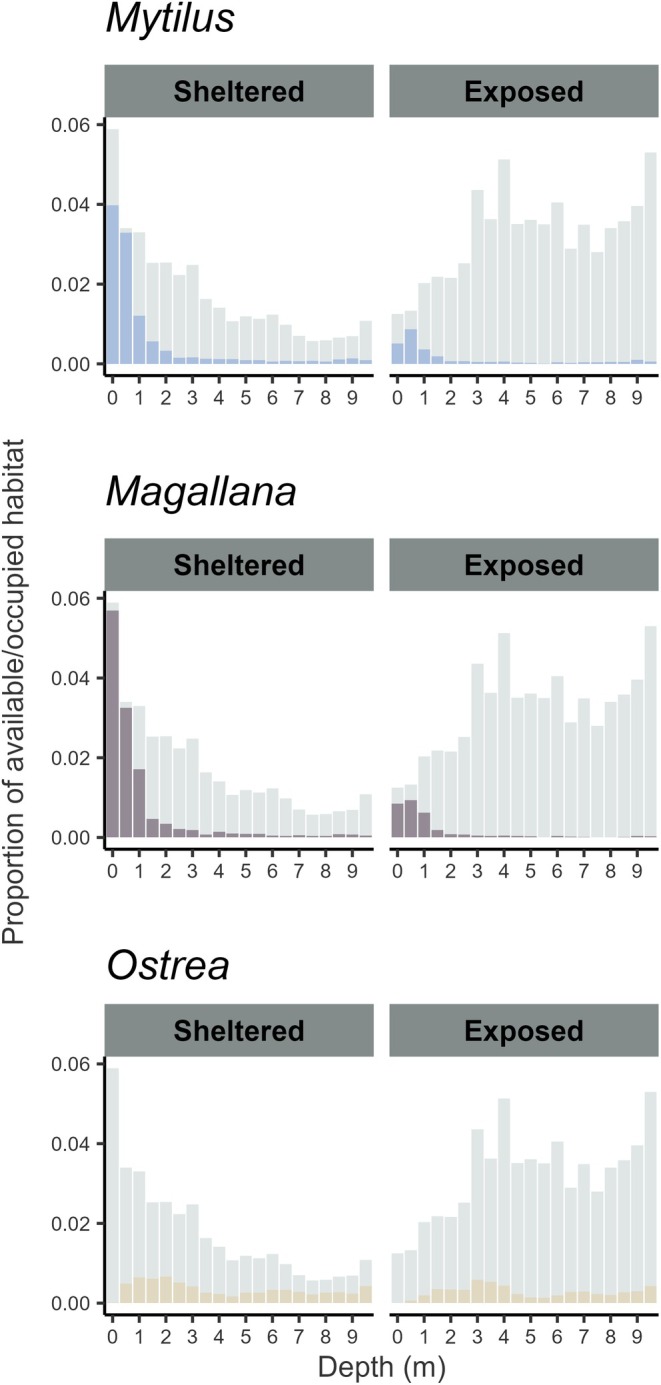
The proportion of available habitat (gray) and occupied (colored) for each species (*Mytilus* (*edulis*): Blue mussel, *Magallana* (*gigas*): Pacific oyster, *Ostrea* (*edulis*): European flat oyster) within each 0.5 m interval between 0 and 10 m depth for sheltered (left) and exposed (right) habitats.

The total area predicted to be occupied by *Mytilus*, *Magallana*, and *Ostrea* was 92, 109, and 84 km^2^, respectively. *Mytilus* was predicted to occupy about 13.2% of the total area (within the study area in depths between 0 and 10 m), 86% of which was shared with *Magallana* and 11% with *Ostrea* (Table 2). *Magallana* was predicted to occupy 15.6% of the total area, 72% of which was shared with *Mytilus* and 8% with *Ostrea*. *Ostrea* was predicted to occupy 12% of the total area, only 13% of which was shared with *Mytilus* and 11% shared with *Magallana*. 72.2% of the total area was predicted to contain none of the species. The percentage of the occupied area in which each species occurred alone was 11%, 26%, and 79% for *Mytilus*, *Magallana*, and *Ostrea*, respectively. In 1.1% of the total area, all three species were predicted to be present. In accordance with the variable importance and partial dependence of depth (see Section 3.4), subsamples from the projection showed that both *Mytilus* and *Magallana* were predicted to be present mostly in the shallowest depths (0–1 m), occupying nearly all available habitat (Figure 7). The occupancy was, however, slightly lower in exposed compared to sheltered habitats (Figure 7). Contrastingly, *Ostrea* was predicted to be completely absent in the shallowest (0–0.5 m) habitats (sheltered and exposed) and most prevalent in deep sheltered habitats, which, however, constituted the least amount of area (Figure 7).

**TABLE 2:** Percentage of predicted occurrence of each species (*Mytilus* (*edulis*): Blue mussel, *Magallana* (*gigas*): Pacific oyster, and *Ostrea* (*edulis*): European flat oyster) and in combination with any of the other two within all the 10 × 10 m grid cells.

Species	%
None	72.2
*Mytilus* only (*T* = 13.2%)	1.5
*Magallana* only (*T* = 15.6%)	4.1
*Ostrea* only (*T* = 13%)	10.3
*Mytilus + Magallana*	10.2
*Mytilus + Ostrea*	0.4
*Magallana + Ostrea*	0.2
*Mytilus + Magallana + Ostrea*	1.1

*Note:* Total percentage of occurrence regardless of the presence of other species (T) is given in brackets.

#### 3.5.2 Total Population Size Estimates

Using the simplest approach of extrapolating the mean abundance across several zones and depth strata (Method 1), the total population of *Mytilus*, *Magallana*, and *Ostrea* was estimated at 1991, 421, and 47 million respectively (Table 3). Incorporating the presence–absence models into the estimation (Method 2) changed these estimates marginally, with the total population of 2080, 549, and 42 million of *Mytilus*, *Magallana*, and *Ostrea* respectively. Finally, estimates from the combination of presence–absence and abundance models (Method 3) resulted in reduced estimates for *Mytilus* (620 million) and *Magallana* (241 million), but similar for *Ostrea* (45 million). Across all methods, *Mytilus* came out as the most abundant species, followed by *Magallana* and *Ostrea* as the least abundant. However, when estimates from Method 3 were converted into total biomass (dry weight), *Magallana* had the largest weight at 557 t, followed by *Mytilus* at 490 t and *Ostrea* with 67 t (Table 3).

**TABLE 3:** Populations size estimates (millions of individuals) (±SE) of three methods using spatially implicit modeling (Method 1), presence–absence modeling (Method 2), and presence–absence combined with abundance modeling (Method 3) for each species (*Mytilus* (*edulis*): Blue mussel, *Magallana* (*gigas*): Pacific oyster, and *Ostrea* (*edulis*): European flat oyster). Dry weight and live wet weight biomass estimates (metric tonnes) (± SE) are based on the results from Method 3 and average weights of each species.

Species	Method 1	Method 2	Method 3	Dry weight	Live wet weight
*Mytilus*	2000 ± 290	2080 ± 78	620 ± 30	490 ± 33	12,700 ± 860
*Magallana*	420 ± 43	550 ± 51	214 ± 6	560 ± 30	31,000 ± 1800
*Ostrea*	47 ± 8	43 ± 3	45 ± 2	67 ± 9	2600 ± 320

#### 3.5.3 Patterns of Abundance

**FIGURE 8:**
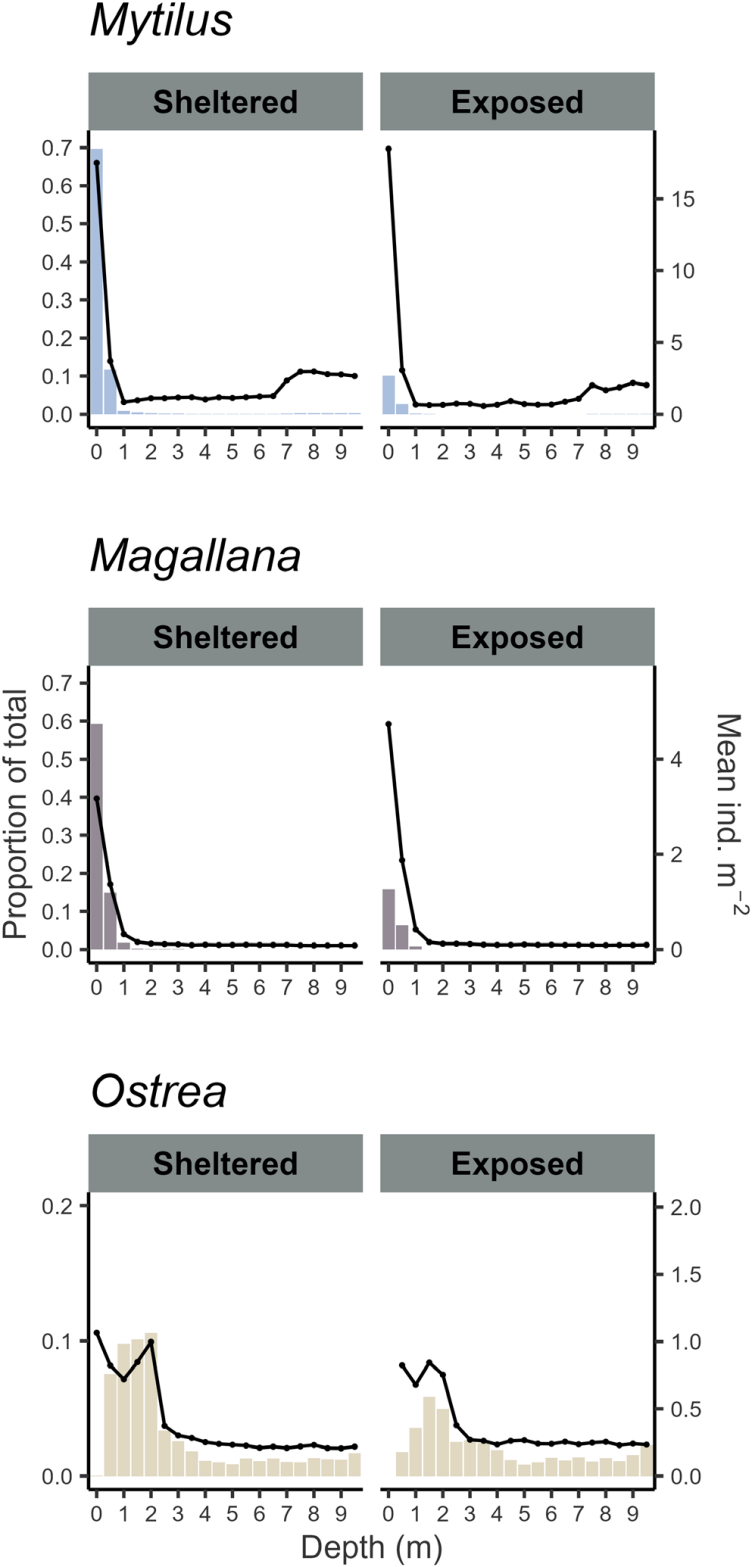
The proportion of total abundance (left axes, colored bars) and mean abundance (ind. m‐2) (right axes, lines) of the modeled population for each species (*Mytilus* (*edulis*): Blue mussel, *Magallana* (*gigas*): Pacific oyster, *Ostrea* (*edulis*): European flat oyster) within each 0.5 m interval between 0 and 10 m depth for sheltered (left) and exposed (right) habitats.

Stratification on depth and exposure for the total modeled abundances showed similar patterns to those of occupancy (Figure 8) but was more pronounced. Mean abundances were considerably higher in the shallowest habitats for both *Mytilus* and *Magallana*. *Ostrea* was found throughout all habitats deeper than 0.5 m, while being completely absent from the shallowest regions. Its highest mean abundance was found between 0.5 and 2.5 m, after which it sharply declined and remained low down to 10 m. Overall, 80% of *Mytilus* were predicted to reside between 0 and 0.5 m deep, 86% of which were in sheltered habitat and 14% in exposed. 80% of *Magallana* were predicted to reside between 0 and 0.6 m deep, 80% of which were in sheltered habitat and 20% in exposed. 80% of *Ostrea* were predicted to reside between 0.6 and 6.0 m deep, 61% of which were in sheltered habitat and 39% in exposed.

**FIGURE 9:**
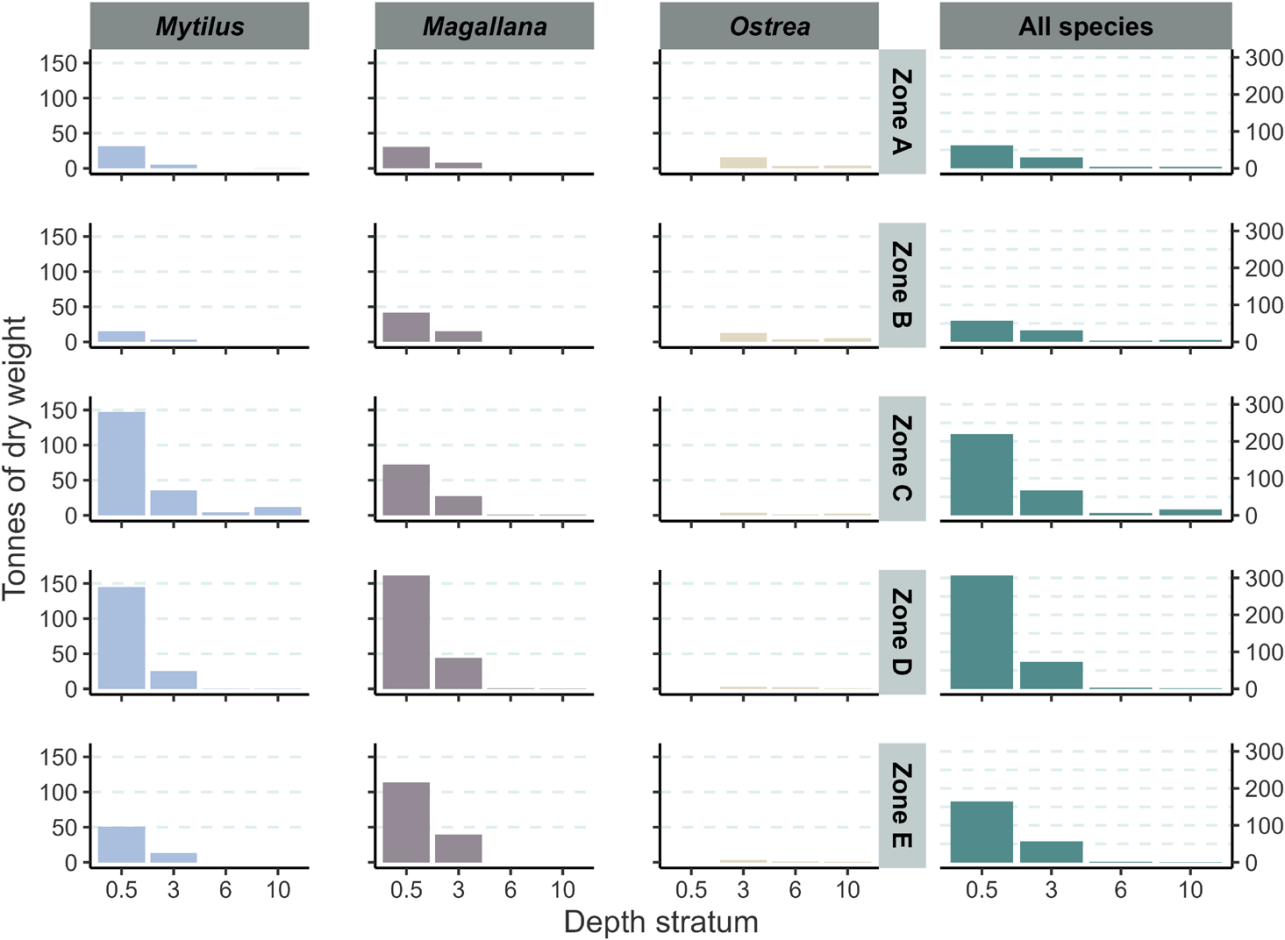
The estimated tonnes of dry weight (DW, from Method 3) of each species (*Mytilus* (*edulis*): Blue mussel, *Magallana* (*gigas*): Pacific oyster, *Ostrea* (*edulis*): European flat oyster) and all species combined (columns) across four depth strata (0–0.5, 0.5–3, 3–6, and 6–10) within each zone (rows, A–E).

When comparing the total biomass across the zones of the study area, each species showed a different large‐scale distribution pattern (Figure 9). The largest part of the *Mytilus* population was predicted to be in the inner section of the fjords around the islands of Tjörn and Orust (zones C and D). The *Magallana* biomass was predicted to be distributed more evenly along the entire coast but was nevertheless particularly high in the south (zones D and E). The majority of the *Ostrea* population was predicted to occur in the northern parts of the coastline (zones A and B).

## 4 Discussion

In this study, we explored the use of a two‐step modeling approach for assessing habitat use, population sizes, and relative ecological importance of three epifaunal marine bivalve species (Blue mussel *M. edulis* , Pacific oyster *Magallana gigas*, and European flat oyster *O. edulis* ) within a stretch of coast that represents the core distribution area of *Magallana* and *Ostrea* in Sweden. Well‐performing abundance models lowered estimated population sizes for *Mytilus* and *Magallana* substantially, while the poor model for *Ostrea* made little difference. Several interesting patterns in distribution and abundance were observed, with both overlap and differences among species distributions. These results further our understanding of the relative ecological importance of each species in different areas and habitat types, as well as demonstrate the usefulness of combining SDMs with models of abundance for answering ecological questions.

### 4.1 Species Level Interactions

The performance of the presence–absence models was adequate for all three species (AUC ≈ 0.8, Figure 3), but noticeably lower than previous models on *Magallana* and *Ostrea* (Bergström et al. 2021). This may be a result of the larger areal extent of this study combined with a lower density of sampled sites compared to the other studies. Moreover, several variables which are known to impact bivalve physiologically from literature (e.g., Chlorophyll concentration, SPM, salinity, and temperature; Kamermans et al. 2013; Beukema et al. 2017; Sanders et al. 2018; Saulsbury et al. 2019) were, in this study, not the most impactful for the presence–absence and abundance models. Although important from a biological perspective, these variables were potentially not variable enough within the study area and may be more important in areas with steeper environmental gradients or on a local scale analysis. Nevertheless, the model predictions of bivalve occurrences were highly consistent among the bootstrapped iterations for *Mytilus* and *Magallana* (Figure 4), likely being driven by the strong depth preference of the two species (Table 3). Similarly, the abundance models performed reasonably well for *Mytilus* and *Magallana* (Figures 5 and 6) and displayed similar performance metrics to models in other studies (Oppel et al. 2012; Rullens et al. 2021; Waldock et al. 2022). For *Ostrea*, all models, however, performed poorly, likely because of the apparent weaker habitat preferences of *Ostrea* and the fact that the prevalence of *Ostrea* in the field data was substantially lower compared to the other species.

*Mytilus* and *Magallana* displayed similar patterns of both habitat use and of abundance at different depths and wave exposure levels (Figures 7 and 8), resulting in a high degree of overlap (Table 2) and potentially significant species interactions. On the other hand, large scale geographical distribution and abundance patterns differed between the species (Figure 9). *Mytilus* was most abundant in zones C and D, where it was mainly concentrated within the inner‐fjord systems located in this area (Figure S3). This is consistent with findings of high larval retention and genetic differentiation of mussels in this area compared to in the rest of the study area (Gustafsson et al. 2024). *Magallana* was slightly more evenly distributed, but with the highest abundances in the zones D and E. As suggested by the general invasion pattern, there is typically a lag and expansion phase before an invasive species reaches equilibrium with the invaded environment during the persistence phase (Geburzi and McCarthy 2018). Consequently, given the recent invasion of *Magallana* in the studied region (since 2006, Wrange et al. 2010), *Magallana* may not yet have fully realized its ecological niche. The results from this study may hence serve as a current baseline for assessing future changes in both population distribution and size, as well as of habitat use overlaps between, *Mytilus* and *Magallana*. Previous research has indicated complex dynamics involving both competitive (e.g., for space and food; Diederich 2005; Joyce et al. 2021) and facilitating interactions (e.g., giving protection against predators and settlement substrate; Reise et al. 2017; Troost et al. 2009) between the species in other regions. There are, however, strong indications that co‐existence is both feasible and likely (Diederich 2005; Troost et al. 2009; Troost 2010; Reise et al. 2017). Nevertheless, the possibility to assess future population‐ and habitat overlap expansion of both *Mytilus* and *Magallana* may provide new and valuable insights for management of the species in the region.

The geographic and spatial distribution patterns of *Ostrea* were very different compared to the other species (Figures 7 and 9), and thus its distributional overlap with the other species was small (Table 2). The highest occupancy of *Ostrea* was observed in the deepest sheltered habitats. However, these habitats were relatively rare and contributed little to the overall area. Therefore, the similarity in area of occupied habitat by *Ostrea* (13%) compared to the other two species (*Mytilus* 13.2%, *Magallana* 15.6%) can mainly be explained by its relatively higher occupancy in common habitats such as deep and exposed areas (Figure 7), though the predicted abundances therein were low (Figure 8). Additionally, *Ostrea* was mostly concentrated in the north in zones A and B, and in zone A was even estimated to contribute the most to the total bivalve biomass in the 0.5–3 m stratum, despite the presence of the other two species at these depths. This differentiation in habitat use and geographical distribution between *Ostrea* and the other species suggests that ecological interaction between the species may be less extensive than previously thought. Nevertheless, it was observed that all three species used each other's living and dead shells as settlement substrate, as reported by Christianen et al. (2018). Thus, there is interaction between the species, though the exact implications of which are not fully understood to date. In concurrence with the indication of limited interactions, no significant temporal changes in the number of *Ostrea* in zones A–C were observed between this study (35.8 million individuals) and 2013–2014 (36.6 million individuals, Thorngren et al. 2019), indicating that the *Ostrea* population has remained stable in the area and highlighting the robustness of the modeling approach as the models were developed on completely different datasets. This study, however, also provides the first population estimate of this vital remnant population across the entire occupied range in Sweden and can serve as a current baseline for monitoring its development in the future.

### 4.2 Relative Importance for Ecosystem Functions

In this study, three methods with varying levels of inclusion of modeling to generate population size estimates were evaluated. The three methods for extrapolation of population sizes, ranging from simple (mean extrapolation only) to more advanced (extrapolation based on occurrence and abundance models), showed varying effectiveness depending on species. Little variation between methods was observed for *Ostrea*, for which the developed models also performed worse compared to the other species, while the extrapolated population sizes were lower for *Mytilus* and *Magallana* using the most advanced method. Modeling species abundance is particularly challenging, as patterns in abundance appear to be more erratic than occurrences. Nonetheless, in this study, the abundance model of both *Mytilus* and *Magallana* produced different results for population size estimates compared to methods without modeling, while poorly performing models (i.e., for *Ostrea*) gave similar results (Table 3). This indicates that not only did the abundance models result in more realistic estimates of population size despite moderate model performance, but also that a poorly fitting model did not give unrealistic predictions. Inclusion of biotic factors (e.g., predation, pathogens, or dispersal patterns) has been shown to improve the performance of distribution models (Wisz et al. 2013; Leach et al. 2016), hence, one way to further improve the estimations of population sizes of the studied species in the area may be to include biotic factors in future modeling.

Estimations of species population sizes within a region can give important insights for various applications such as identification of critical species for ecological processes and quantification of ecosystem services (e.g., zu Ermgassen et al. 2013), attribution of status of concern for threatened species (Powles et al. 2000), and assessment of cost‐effectiveness of invasive species management (Yokomizo et al. 2009). Overall, the abundance of *Mytilus* was estimated to be approximately 3 times larger than that of the non‐native *Magallana* and 10–50 times more abundant than *Ostrea* for all three methods (Table 3). However, when converted to more ecologically relevant estimates of biomass, the situation was reversed and the biomass of *Magallana* was estimated to be approximately 3 times larger than that of *Mytilus* (Table 3). These findings are consistent with a recent survey from a more limited area in the northern part of this study area (Greeve et al. 2023) and suggest that *Magallana* currently contribute more to ecosystem functions which are directly related to biomass (e.g., filtration) than the other two species in shallow habitats. Recently, concerns have been raised about the decline of wild *Mytilus* populations in the study area (Baden et al. 2021), and in this context, the expansion of the non‐native *Magallana* in habitats where *Mytilus* used to be present may compensate for some lost ecosystem functions from reduced *Mytilus* populations.

Not all functions are, however, directly replaceable between species. Numerous studies have compared the autogenic ecosystem engineering properties of *Magallana* to those of native epibenthic bivalves (e.g., impacts on benthic community structure and diversity; Kochmann et al. 2008; Green et al. 2013; Hollander et al. 2015; Norling et al. 2015, Zwerschke et al. 2016, 2018; Guy et al. 2018; Andriana et al. 2020). Impacts seem to be mediated by both the abundance of *Magallana* and local environmental conditions, making generalizations across large spatial scales difficult (Herbert et al. 2016). Given the large separation in habitat preferences between *Ostrea* and the other bivalve species, *Ostrea* is likely to be the main contributor of essential functions in the 3–6 m depth range (Figure 9), and even in some shallower habitats in the northern part of the study area, limited additions of *Magallana* may act to enhance ecosystem functions as community structure associated with *Magallana* has been observed to be similar to that of *Ostrea* (Zwerschke et al. 2020). In contrast, *Mytilus* and *Magallana* contribute to different community structures (Kochmann et al. 2008; Norling et al. 2015) concentrated in a very narrow depth range within or just below the intertidal zone and mostly within sheltered habitats. Consequently, as the *Magallana* population is expanding (Laugen et al. 2015), and as the *Mytilus* population is suggested to be in decline (Baden et al. 2021), a shift in species composition may be expected in the intertidal zone.

### 4.3 Implications for Management

As indicated in the previous sections, SDMs may be valuable tools in supporting conservation and management efforts, which is also confirmed by other studies in both terrestrial and aquatic environments (Lindegarth et al. 2014; Rullens et al. 2022; Panzeri et al. 2024). For instance, they can aid in deciding whether intervention is needed to preserve a species' natural range, or the expected outcomes of (re)introductions of native and invasive species (Linde et al. 2012; Lake et al. 2020). In concurrence, this study applied SDM and quantitative abundance modeling to provide a basis for informed management of wild epibenthic bivalves in Sweden and to meet knowledge gaps identified jointly between management authorities and academia, highlighting a multi‐actor approach to management. The estimated population sizes of native bivalves fill fundamental knowledge gaps by providing a current baseline of how much, and where, epibenthic bivalves are found. Such information is becoming increasingly crucial for the monitoring and management of marine ecosystems and biodiversity under threat from expanding anthropogenic impacts (Estes et al. 2021). Moreover, the high‐resolution modeling approach applied further enhances the applicability of the results by enabling the identification of areas of particular interest for restoration work or hotspots for creating marine protected areas (Sundblad et al. 2011; Ferrari et al. 2018), aspects of increasing importance since the adoption of the new Restoration law in 2024 by the European Union (EU 2024). If coupled with oceanographic trajectory modeling and biophysical models, the high‐resolution maps produced can also aid in identifying important hotspots (and gaps) to maintain connectivity between valuable bivalve reefs (Berkström et al. 2022). In addition to being beneficial to native species management, the models also infer advantages related to invasive species management and understanding of species interactions. The mapping of the invasive *Magallana* in this study provides important insights into large‐scale geographical distribution patterns and thereby facilitates the identification of areas of high importance for management, e.g., high‐density locations where removal or other management efforts could be directed. Although complete eradication of *Magallana* is not feasible in its established range, there are examples of small‐scale removal efforts (Hansen et al. 2023), effectively mitigating the most pronounced impacts of the species.

Finally, production of extractive species such as mussels and oysters may enhance the occurrence and abundance of wild bivalves through larval spillover (Norrie et al. 2020; Delago 2021). This is in concurrence with the results from this study, where a large proportion of the *Mytilus* population was found to be centered in inner fjord systems of zones C and D, as these waters also harbor the majority of mussel aquaculture production in Sweden (Bergström et al. 2024). In fact, the yearly mussel production in the area corresponds to approximately 17% of the total modeled wild population, meaning a substantial part of the total *Mytilus* stock resides in these aquaculture operations. Wild *Mytilus* in the area have also been demonstrated to be genetically and oceanographically isolated from other areas, suggesting that most larvae released are retained within the fjords (Gustafsson et al. 2024). It is, however, possible that the high prevalence of both wild and farmed mussels in the area merely reflects suitable habitat conditions; hence, the possible restorative properties of mussel aquaculture in the area should be evaluated further before any general conclusions can be made. If larval spill over can be confirmed to contribute to the occurrence and abundance of wild mussel populations, well‐placed (with regard to dispersal patterns) aquaculture systems may have the potential to be used as a tool to enhance and restore wild native bivalve beds of both *Mytilus* and *Ostrea* in Sweden, in concurrence with the concept of restorative aquaculture (The Nature Conservancy 2021). In contrast to *Mytilus*, aquaculture production of *Ostrea* is, to date, very limited in Sweden (1–3 t on a yearly basis), and most production is from harvested wild populations (3–7 t). Aquaculture of *Magallana* is not allowed due to its recent establishment, but commercial harvest of wild populations has commenced and is increasing (from 2 t in 2013 to 9 t in 2020–2023, data from the Swedish Food Authority). Based on the production numbers and the modeled population sizes, the current commercial use of wild populations of both species constitutes < 0.1% and < 0.001% for *Ostrea* and *Magallana*, respectively. For *Ostrea*, a precautionary approach in terms of harvest is warranted given the unique status of the population, being one of the largest in Europe (Thorngren et al. 2019), but for *Magallana*, being an invasive species, increased harvest would only be beneficial.

### 4.4 Methodological Considerations

Evaluating the performances of presence–absence and abundance models is an important step in assessing the realisms of the produced predictions (Elith and Leathwick 2009). However, as the field of SDMs is progressing, researchers are becoming more aware of the challenges in properly representing uncertainties (Barry and Elith 2006; Beale and Lennon 2012). The bootstrap 0.632+ method used in this study provided an efficient and easily implemented protocol for producing near‐unbiased estimates of a variety of performance metrics while using the available data optimally. Additionally, it can be used to both estimate important parameters that are used in the prediction phase (e.g., the cut‐off threshold) and generate the uncertainties around them and the resulting prediction. Furthermore, extrapolating beyond the sampled area, whether spatially or in relation to changing variables (e.g., climate change scenarios), is a common source of uncertainty in SDM studies (Elith and Leathwick 2009; Brodie et al. 2022), but this issue was largely not relevant to this study. Therefore, the reported model performances can be considered to accurately reflect the true model performance.

The choice of models used for presence–absence and abundance models (gradient boosting classification/regression trees) was primarily based on the predictive power and ability to handle complex data structures (Elith et al. 2008). They are, however, not ideal for making inferences on the ecological importance of variables and thus not a preferred choice when assessing a species' niche (Tredennick et al. 2021), a disadvantage further corroborated by using moderately correlated variables (VIF = 1–5) such as exposure and the in‐ offshore position (X) in this study. The inclusion of these variables was believed to have produced better predictions yet introduced problems with assessing the importance of these variables for the models separately. This issue was, however, circumvented by assessing and inferring from the distribution and abundance patterns observed in the predictions (Figures 7, 8, 9). Nonetheless, it was not possible, nor was it the aim, to fully understand the underlying mechanisms for the resulting predictions.

### 4.5 Conclusion

Empirical models of species and habitat distributions in the marine environment typically focus on occurrence rather than quantitative aspects such as abundance and biomass (but see Oppel et al. 2012; Rullens et al. 2021; Stephenson et al. 2023; Young and Carr 2015 for exceptions). This is unfortunate since the importance of ecological functions and ecosystem services can be expected to be more closely related to quantities of ecosystem components rather than just the mere presence. In this study, we demonstrate that the combination of presence–absence models and models of abundance can be used to improve estimated population sizes more accurately and precisely than from simple upscaling of mean abundances (e.g., Greeve et al. 2023; Thorngren et al. 2019), and to support analysis of habitat use and habitat overlap between species. This allowed for the evaluation of the relative ecological importance of epifaunal bivalve species within the study area and in the different habitats within. It was observed that the invasive *Magallana* contributes the most to the total epifaunal bivalve biomass, and thus likely also to biomass‐linked ecosystem functions and services, in shallow, sheltered habitats, but that the native *Ostrea* was important in deeper and more exposed areas. The abundance models enhanced the functionality of the projections for application in the management of both native and invasive species, as exemplified in a number of cases. The methods described here can be replicated and adapted to studying other species in other areas although improvements in handling uncertainties in model prediction in SDMs are needed. To further improve knowledge on epifaunal bivalves on the Swedish west coast, more work should be directed toward methods to accurately describe factors driving patterns in occurrence and abundance, and data collection to improve model performance and monitor changes in population size and distribution should be continued.

## Author Contributions

**Youk Greeve:** conceptualization (equal), data curation (lead), formal analysis (lead), methodology (lead), visualization (lead), writing – original draft (lead), writing – review and editing (equal). **Molly C. Reamon:** investigation (equal), methodology (supporting), writing – review and editing (equal). **Per Bergström:** data curation (supporting), writing – review and editing (equal). **Åsa Strand:** conceptualization (equal), investigation (equal), writing – original draft (supporting), writing – review and editing (equal). **Ane T. Laugen:** investigation (equal), writing – review and editing (equal). **Mats Lindegarth:** conceptualization (equal), writing – original draft (equal), writing – review and editing (equal).

## Conflicts of Interest

The authors declare no conflicts of interest.

## Supporting information


**Appendix S1:** ece372586‐sup‐0001‐AppendixS1.docx.

**Table S3:** List of predictors used to model and predict occurrence and abundance of Mytilus, Magallana and Ostrea.

## Data Availability

Raw data used for the models are available through the Open Science Framework https://osf.io/4ft8g/?view_only=d35ee44794484116b4841209a112524e.
