# Supplementary material for: Improving Large‐Scale Population Estimates and Assessments of the Ecological Importance of Three Epifaunal Bivalve Species by Combining Distribution and Abundance Models
Source: Ecol Evol. 2025 Dec 8;15(12):e72586. doi: 10.1002/ece3.72586 (PMC12683372; doi:10.1002/ece3.72586)
Supplement: Supplementary file 1 — Appendix S1: ece372586‐sup‐0001‐AppendixS1.docx. [file ECE3-15-e72586-s002.docx]

Appendix for:

**Improving large-scale population estimates and assessments of ecological importance of three epifaunal bivalve species by combining distribution and abundance models**

Youk Greeve^1^*, Molly C. Reamon^3^, Per Bergström^1^, Åsa Strand^2^, Ane T. Laugen^3^ and Mats Lindegarth^1^

^1^ Department of Marine Sciences - Tjärnö

University of Gothenburg,

Tjärnö, SE-452 96 Strömstad, Sweden

^2^ Department of Enviromental Intelligence

IVL Swedish Environmental Research Insitute

Kristineberg 566

SE-451 78 Fiskebäckskil, Sweden

^3^Department of Natural Sciences, Centre for Coastal Research (CCR)

University of Adger

NO-4604 Kristiansand, Norway

Corresponding author: Youk Greeve

Tel: Int. +46 7 66184099

Email: [youk.greeve@gu.se](mailto:youk.greeve@gu.se)

This following part of the supporting information provides a description of each equation used for estimating total population size for Method 1 – 3 and total biomass for Method 3.

Method 1:

The overall mean density ($\bar{y}_{total}$) is calculated through:

$$\begin{aligned} \bar{y}_{total}\text{ = }\sum_{st} w_{st} \times\bar{y}_{st}\#\left( M1 eq. 1 \right) \end{aligned}$$

Where $w_{st}$ is the weight (areal proportion) and $\bar{y}_{st}$ is the estimated mean density for each depth stratum within each zone.

The total variance ($V_{\bar{y}_{total}}$) is calculated through:

$$\begin{aligned} V_{\bar{y}_{total}}= \sum_{st} \frac{w_{st}^{2} \times\sigma_{st}^{2}}{n_{st}} \#\left( M1 eq. 2 \right) \end{aligned}$$

Where $\sigma_{st}^{2}$ is the standard deviation and $n_{st}$ is the number of samples taken for each depth stratum within each zone.

The total population size and the error margins (SE) are calculated through:

$$\begin{aligned} \bar{y}_{total} \times N \pm\sqrt{V_{\bar{y}_{total}}} \times N \#\left( M1 eq. 3 \right) \end{aligned}$$

Where $N$ is the total areal extent of the study area (m^2^).

Method 2:

This method is an adaptation to incorporate results from the presence-absence model and the variation introduced by the variation around the estimate of the optimal cut-off threshold. The overall mean density ($\bar{y}_{total}$) is calculated through:

$$\begin{aligned} \bar{y}_{total}\text{ = }\sum_{st} w_{st} \times\bar{y}_{st}\times\bar{p}_{st}\#\left( M2 eq. 1 \right) \end{aligned}$$

Where in this method $\bar{y}_{st}$ is estimated only from sites where the species is present and $\bar{p}_{st}$ is the mean proportion (of all bootstrapped cut-off thresholds) of the area that it is predicted to be present.

The variances associated with $\bar{y}_{st}$ and $\bar{p}_{st}$ ($V_{\bar{p}_{st}/\bar{y}_{st}}$) are calculated with:

$$\begin{aligned} V_{\bar{p}_{st}/\bar{y}_{st}}= \frac{w_{st}^{2} \times\sigma_{\bar{p}_{st}/\bar{y}_{st}}^{2}}{n_{st}} \#\left( M2 eq. 2 \right) \end{aligned}$$

And the total variance ($V_{\bar{y}_{total}}$) is calculated through propagation of error:

$$\begin{aligned} V_{\bar{y}_{total}} = \sum_{st} {(w}_{st} \times\bar{y}_{st}\times\bar{p}_{st})\sqrt{\left( \frac{V_{\bar{p}_{st}}}{\bar{p}_{st}} \right)^{2}+\left( \frac{V_{\bar{y}_{st}}}{\bar{y}_{st}} \right)^{2}}\#\left( M2 eq. 3 \right) \end{aligned}$$

The total population size and the error margins (SE) are again calculated through:

$$\begin{aligned} \bar{y}_{total} \times N \pm\sqrt{V_{\bar{y}_{total}}} \times N \#\left( M2 eq. 4 \right) \end{aligned}$$

Method 3

In method 3 population sizes were estimated by incorporating the result from the abundance model and the presence-absence model. Population size estimates were produced for each cut-off threshold, which was used to delimit the abundance model layer. Population size estimates were calculated as:

$$\begin{aligned} \frac{\bar{P}_{total}}{\bar{r}} \#\left( M3 eq. 1 \right) \end{aligned}$$

Where $\bar{P}_{total}$ is the mean of population size estimates for all cut-off threshold (n= 100) and $\bar{r}$ is the mean ratio (or bias) of each bootstrap iteration of the abundance model, which is calculated as:

$$\begin{aligned} \bar{r} = \frac{\sum Pred}{\sum Obs} \#\left( M3 eq. 2 \right) \end{aligned}$$

The error margins around the population size estimate were calculated as:

$$\begin{aligned} \frac{\bar{P}_{total}}{\bar{r}} \times\sqrt{\left( \frac{\sigma_{\bar{P}_{total}}}{\bar{P}_{total}} \right)^{2}+\left( \frac{\sigma_{\bar{r}}}{\bar{r}} \right)^{2}} \#\left( M3 eq. 3 \right) \end{aligned}$$

For total biomass, the following formula was used:

$$\begin{aligned} \frac{\bar{P}_{total}}{\bar{r}} \times\bar{BM} \#\left( M3 eq. 4 \right) \end{aligned}$$

Where $\bar{BM}$ is either the mean dry weight biomass or live wet weight of individual species. The error margins around the total biomass estimates were calculated as:

$$\begin{aligned} \left( \frac{\bar{P}_{total}}{\bar{r}} \times\bar{BM} \right) \times\sqrt{\left( \frac{\sigma_{\bar{P}_{total}}}{\bar{P}_{total}} \right)^{2}+\left( \frac{\sigma_{\bar{r}}}{\bar{r}} \right)^{2}+\left( \frac{\sigma_{\bar{BM}}}{\bar{BM}} \right)^{2}} \#\left( M3 eq. 5 \right) \end{aligned}$$

*Table A1. List of data sets from 2018-2022 included in this study.*

| **Year** | **Geographic scope** | **Video transects** | **Quadrates** | **Source** |
| --- | --- | --- | --- | --- |
| 2018 | Area D | 97 | 0 | Laugen et al. 2023, s.2.2.2 |
|  | Area A | 33 | 0 | " |
| 2019 | Area D | 44 | 32 | " |
|  | Area A | 0 | 16 | " |
|  | Area A | 63 | 0 | " |
| 2020 | Areas B, C and D | 0 | 38 | " |
|  | Area B, C and D | 144 | 0 | " |
|  | Area B, C and D | 144 | 0 | " |
|  | Area D | 7 | 0 | Laugen et al. 2023, s.2.3.1 |
| 2021 | Area A | 0 | 40 | Greeve et al. 2023 |
| 2022 | Area A | 0 | 30 | Greeve et al. unpublished |
| 2023 | Area D | 83 | 85 | Hedensjö et al. 2024 |

*Table A2. Number of presences and absences in the field surveys as well as the mean abundance when present for each species. Dry weight (DW) and live wet weight (WW) are the averages for an individual (sample size given). Error margins are given in standard errors and are calculated from 1000 bootstrap iterations.*

| **Species** | **Absences** | **Presences** | **Mean abundance (ind. m^-2^)** | **Mean DW/WW (g ind.^-1^)** |
| --- | --- | --- | --- | --- |
| *Mytilus* | 516 | 280 | 22.9 ± 4.1 | 0.79/20.4 ± 0.04/0.97, n = 940 |
| *Magallana* | 482 | 314 | 4.8 ± 0.62 | 2.60/145.3 ± 0.12/7.38, n = 605 |
| *Ostrea* | 666 | 130 | 0.7 ± 0.13 | 1.48/57.7 ± 0.19/6.48, n = 73 |

*Table A3. List of predictors used to model and predict occurrence and abundance of Mytilus, Magallana and Ostrea.*


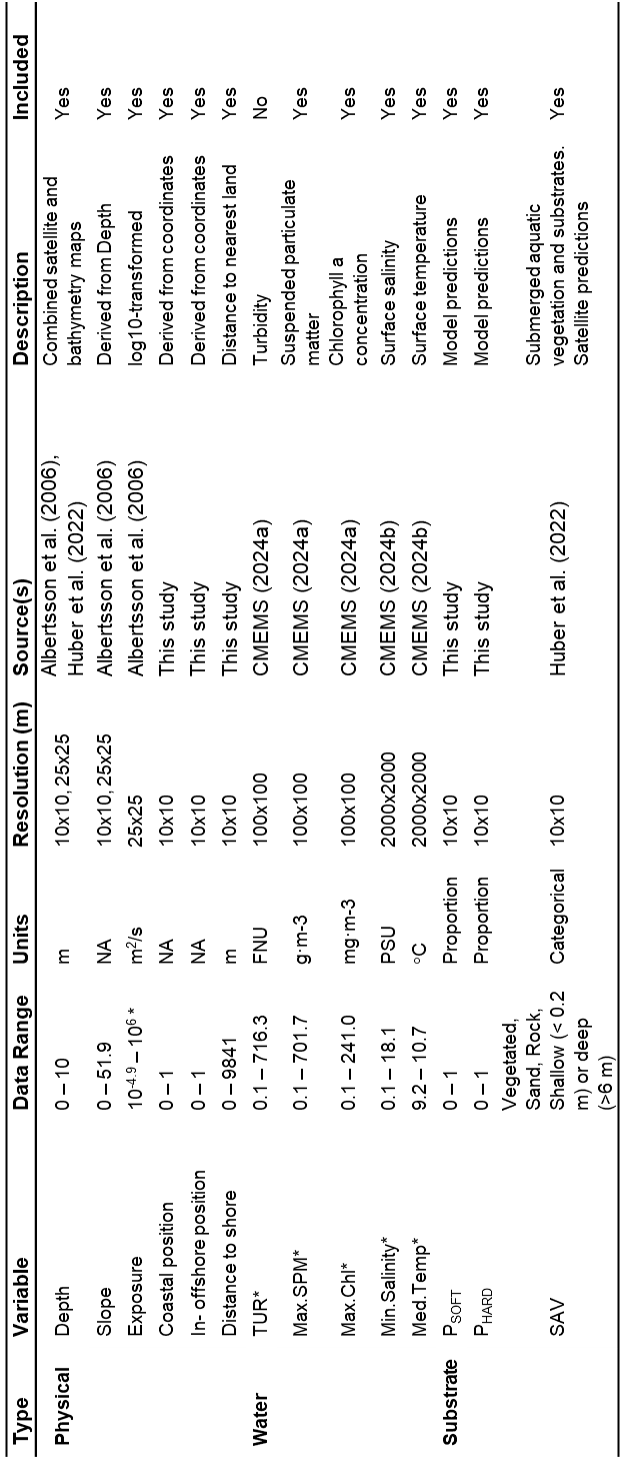


* Monthly maximum, minimum, mean and median satellite data from 2021-2023 were initially assessed.


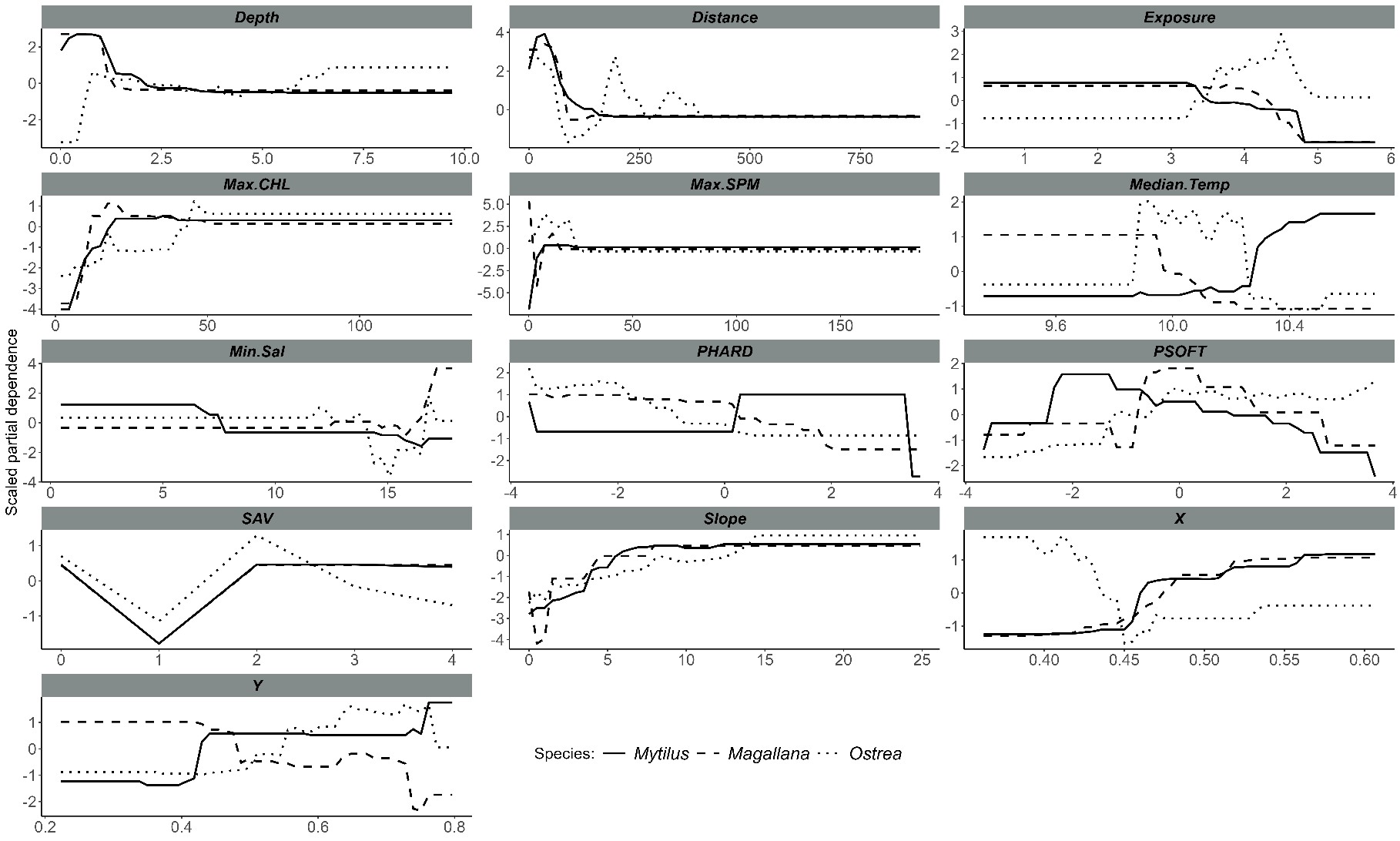


*Figure A1: The partial response curves for each of the variables used for the presence-absence models. Line types indicate the responses for individual species. Categorical levels of the SAV variable are: 0) “Deep”, 1) “Sand”, 2) “Submerged Vegetation”, 3) “Rock”, 4) “Intertidal”.*


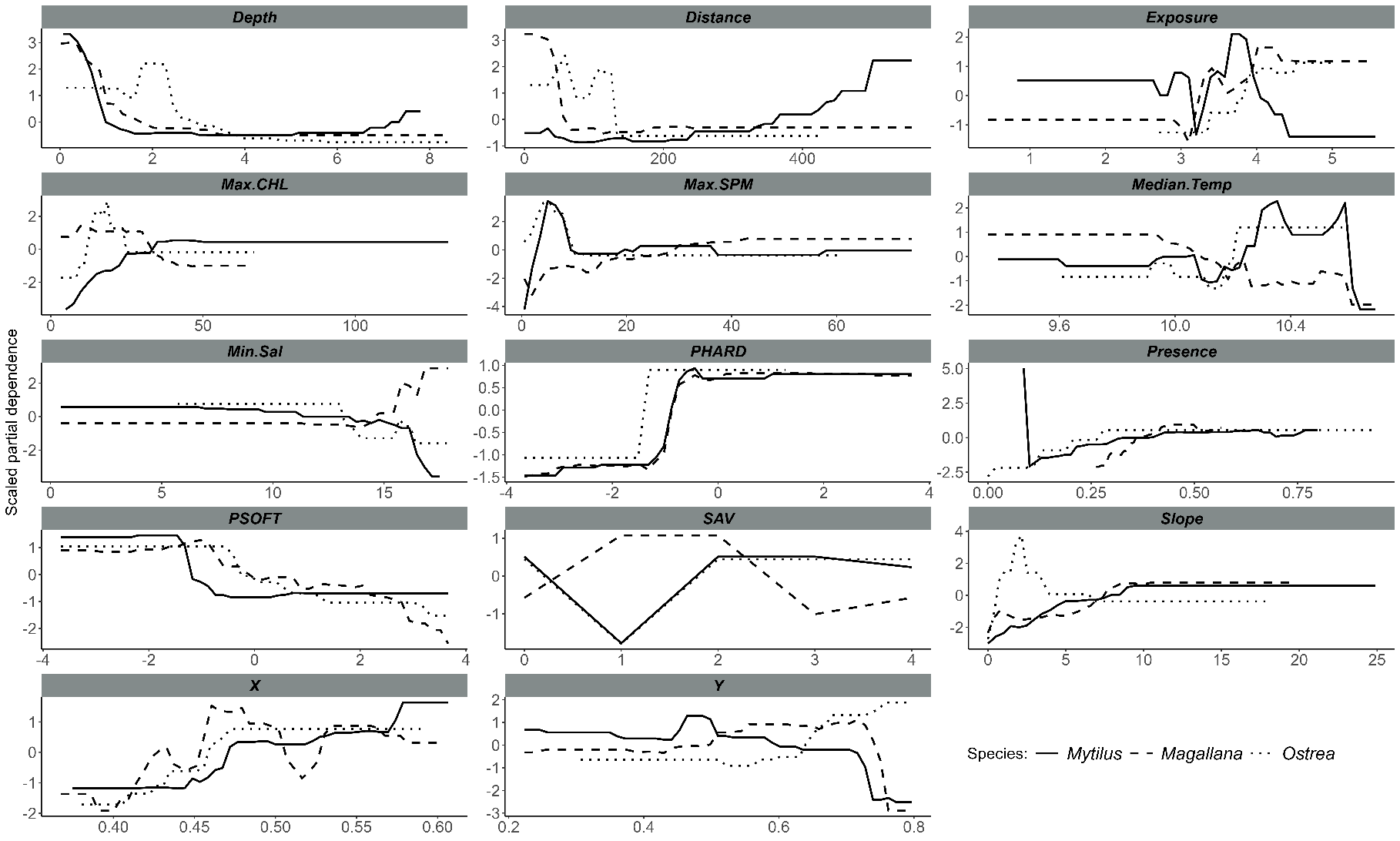


*Figure A2: The partial response curves for each of the variables used for the abundance models. Line types indicate the responses for individual species. Categorical levels of the SAV variable are: 0) “Deep”, 1) “Sand”, 2) “Submerged Vegetation”, 3) “Rock”, 4) “Intertidal”.*


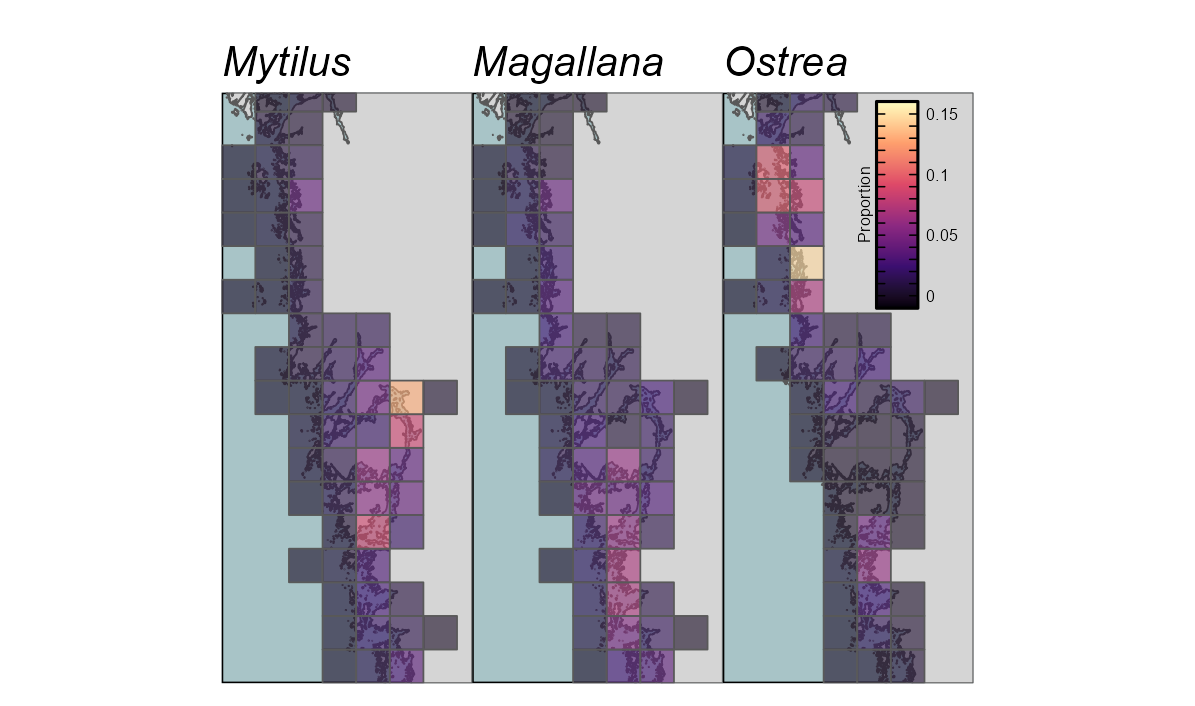


*Figure A3: Proportional contribution of each grid cell to the total modelled population of each species (Mytilus (edulis): blue mussel, Magallana (gigas): Pacific oyster, Ostrea (edulis): European flat oyster). Each cell of the grid is 10 x 10 km.*
