# Supplementary material for: Improving Large‐Scale Population Estimates and Assessments of the Ecological Importance of Three Epifaunal Bivalve Species by Combining Distribution and Abundance Models
Source: Ecol Evol. 2025 Dec 8;15(12):e72586. doi: 10.1002/ece3.72586 (PMC12683372; doi:10.1002/ece3.72586)
Supplement: Supplementary file 2 — Table S3: List of predictors used to model and predict occurrence and abundance of Mytilus, Magallana and Ostrea. [file ECE3-15-e72586-s001.docx]

*Table A3. List of predictors used to model and predict occurrence and abundance of Mytilus, Magallana and Ostrea.*

| **Type** | **Variable** | **Data Range** | **Units** | **Resolution (m)** | **Source(s)** | **Description** | **Included** |
| --- | --- | --- | --- | --- | --- | --- | --- |
| **Physical** | Depth | 0-10 | m | 10x10, 25x25 | Albertsson et al. (2006), Huber et al. (2022) | Combined satellite and bathymetry maps | Yes |
|  | Slope | 0 – 51.9 | NA | 10x10, 25x25 | Albertsson et al. (2006) | Derived from Depth | Yes |
|  | Exposure | 10^-4.9^ – 10^6^ | m^2^/s | 25x25 | Albertsson et al. (2006) | log10-transformed | Yes |
|  | Coastal position | 0 – 1 | NA | 10x10 | This study | Derived from coordinates | Yes |
|  | In- offshore position | 0 – 1 | NA | 10x10 | This study | Derived from coordinates | Yes |
|  | Distance to shore | 0 – 9841 | m | 10x10 | This study | Distance from nearest land | Yes |
| **Water** | TUR* | 0.1 – 716.3 | FNU | 100x100 | CMEMS (2024a) | Turbidity | No |
|  | Max.SPM* | 0.1 – 701.7 | g⋅m^-3^ | 100x100 | CMEMS (2024a) | Suspended particulate matter | Yes |
|  | Max.Chl* | 0.1 – 241.0 | mg⋅m^-3^ | 100x100 | CMEMS (2024a) | Chlorophyll a concentration | Yes |
|  | Min.Salinity* | 0.1 – 18.1 | PSU | 2000x2000 | CMEMS (2024b) | Surface salinity | Yes |
|  | Med.Temp* | 9.2 – 10.7 | °C | 2000x2000 | CMEMS (2024b) | Surface temperature | Yes |
| **Substrate** | P_SOFT_ | 0 – 1 | Proportion | 10x10 | This study | Model predictions | Yes |
|  | P_HARD_ | 0 – 1 | Proportion | 10x10 | This study | Model predictions | Yes |
|  | SAV | Vegetated, Sand, Rock, Shallow (>0.2 m) or deep (>6 m) | Categorical | 10x10 | Huber et al. (2022) | Submerged aquatic vegetation and substrates. Satellite predictions | Yes |

* Monthly maximum, minimum, mean and median satellite data from 2021-2023 were initially assessed.
